# *GCH1* variants contribute to the risk and earlier age-at-onset of Parkinson’s disease: a two-cohort case-control study

**DOI:** 10.1186/s40035-020-00212-3

**Published:** 2020-08-04

**Authors:** Hong-xu Pan, Yu-wen Zhao, Jun-pu Mei, Zheng-huan Fang, Yige Wang, Xun Zhou, Yang-jie Zhou, Rui Zhang, Kai-lin Zhang, Li Jiang, Qian Zeng, Yan He, Zheng Wang, Zhen-hua Liu, Qian Xu, Qi-ying Sun, Yang Yang, Ya-cen Hu, Ya-se Chen, Juan Du, Li-fang Lei, Hai-nan Zhang, Chun-yu Wang, Xin-xiang Yan, Lu Shen, Hong Jiang, Jie-qiong Tan, Jin-chen Li, Bei-sha Tang, Ji-feng Guo

**Affiliations:** 1 Department of Neurology, Xiangya Hospital, Central South University, Changsha, 410008 China; 2 National Clinical Research Center for Geriatric Disorders, Xiangya Hospital, Central South University, Changsha, 410008 China; 3 Centre for Medical Genetics and Hunan Key Laboratory of Medical Genetics, School of Life Sciences, Central South University, Changsha, 410008 China; 4 Department of Geriatrics, Xiangya Hospital, Central South University, Changsha, 410008 China; 5 Department of Neurology, The Third Xiangya Hospital, Central South University, Changsha, 410013 China; 6 Department of Neurology, The Second Xiangya Hospital, Central South University, Changsha, 410011 China; 7 Key Laboratory of Hunan Province in Neurodegenerative Disorders, Central South University, Changsha, 410008 China

**Keywords:** Parkinson’s disease, Age at onset, *GCH1*, Deleterious variants, Non-coding variants

## Abstract

**Background:**

Common and rare variants of guanosine triphosphate cyclohydrolase 1 (*GCH1*) gene may play important roles in Parkinson’s disease (PD). However, there is a lack of comprehensive analysis of *GCH1* genotypes, especially in non-coding regions. The aim of this study was to explore the genetic characteristics of *GCH1*, including rare and common variants in coding and non-coding regions, in a large population of PD patients in Chinese mainland, as well as the phenotypic characteristics of *GCH1* variant carriers.

**Methods:**

In the first cohort of this case-control study, we performed whole-exome sequencing in 1555 patients with early-onset or familial PD and 2234 healthy controls; then in the second cohort, whole-genome sequencing was performed in sporadic late-onset PD samples (1962 patients), as well as 1279 controls. Variants at target *GCH1* regions were extracted, and then genetic and detailed phenotypic data were analyzed using regression models and the sequence kernel association test. We also performed a meta-analysis to correlate deleterious *GCH1* variants with age at onset (AAO) in PD patients.

**Results:**

For coding variants, we identified a significant burden of *GCH1* deleterious variants in early-onset or familial PD cases compared to controls (1.2% vs 0.1%, *P* < 0.0001). In the analysis of possible regulatory variants in *GCH1* non-coding regions, rs12323905 (*P* = 0.001, odds ratio = 1.19, 95%CI 1.07–1.32) was significantly associated with PD, and variant sets in untranslated regions and intron regions, *GCH1* brain-specific expression quantitative trait loci, and two possible promoter/enhancer (GH14J054857 and GH14J054880) were suggestively associated with PD. Genotype-phenotype correlation analysis revealed that the carriers of *GCH1* deleterious variants manifested younger AAO (*P* < 0.0001), and had milder motor symptoms, milder fatigue symptoms and more autonomic nervous dysfunctions. Meta-analysis of six studies demonstrated 6.4-year earlier onset in *GCH1* deleterious variant carriers (*P* = 0.0009).

**Conclusions:**

The results highlight the importance of deleterious variants and non-coding variants of *GCH1* in PD in Chinese mainland and suggest that *GCH1* mutation can influence the PD phenotype, which may help design experimental studies to elucidate the mechanisms of *GCH1* in the pathogenesis of PD.

## Background

Parkinson’s disease (PD) is the second most common neurodegenerative disease characterized clinically by bradykinesia, resting tremor, rigidity, and postural instability. The interplay among aging, genetics and environmental factors plays important roles in PD pathogenesis [1, 2]. The core biological feature of PD is nigrostriatal dopamine deficiency, which is shared by dopa-responsive dystonia (DRD), a disorder characterized by childhood- or adolescent-onset dystonia and dramatic response to low doses of *L*-dopa without degeneration of the nigral neurons [3, 4]. A significant proportion of early-onset PD patients manifest with dystonia [5]; moreover, co-occurrence of DRD and parkinsonism has been reported in families with mutations of guanosine triphosphate cyclohydrolase 1 (*GCH1*) gene [6], which is a well-established disease-causing gene for DRD. GCH1 plays a pivotal role in dopamine biosynthesis [7], indicating that it may also participate in the pathogenesis of PD. Exome-wide analysis has revealed increased burden of predicted deleterious *GCH1* mutations in PD patients [8–10], and genome-wide association studies (GWAS) have identified associations of polymorphisms in *GCH1* locus with PD [11, 12]. However, conflicting results have been reported among different populations [13, 14], indicating the genetic heterogeneity among populations. Thus, it is important to explore whether rare and common *GCH1* variants play a role in PD patients in Chinese mainland. Furthermore, there is still a lack of studies on *GCH1* variants in regulatory regions such as promoter, enhancer, and expression quantitative trait loci (eQTLs). In addition, mutation and polymorphism in the regulatory regions may influence the transcriptional activity by affecting the binding and regulatory ability of transcription factors [15]. Therefore, it is necessary to explore the relationship between variants in *GCH1* regulatory elements and PD.

In addition to the effect on the risk of PD, genetic variants may also play roles in specific clinical phenotypes [16, 17]. With the development of precision medicine, understanding the differences in clinical phenotypes of varied genetic forms of PD may help clinicians in symptomatic treatment and consultation. Previous studies [8–10] have only described specific symptoms of *GCH1* variant-carriers, and there is a lack of systematical analysis of clinical manifestations of *GCH1* variant-carriers. Studies have shown that monogenic genes in PD, such as *PRKN* and *PINK1* [18], and rare damaging variants in PD risk gene such as *GBA* [19], are associated with an earlier age at onset (AAO), which suggested the importance of genetic variants for AAO. Therefore, it is necessary to study the effect of *GCH1* variants on clinical manifestation of PD in our study, especially the AAO.

In the present study, we first analyzed the burden of *GCH1* variants in coding regions in PD patients using whole-exome sequencing (WES), and then examined rare and common variants in the reported regulatory elements of *GCH1* including promoter, enhancer, untranslated regions (UTRs)/introns and eQTLs, using whole-genome sequencing (WGS). Third, we analyzed the clinical manifestations of PD patients with *GCH1* rare or common variants, especially in terms of AAO.

## Materials and methods

### Participants

PD patients of Chinese origin who visited the Department of Neurology, Xiangya Hospital between October 2006 and January 2019 and at other cooperating centers of Parkinson’s Disease & Movement Disorders Multicenter Database and Collaborative Network in China (PD-MDCNC, http://pd-mdcnc.com:3111/) were recruited in the study. They were diagnosed by movement disorder specialists according to the UK brain bank criteria [20] or Movement Disorders Society (MDS) clinical diagnostic criteria [21]. Control participants without any neurological diseases were recruited from community or were the spouse of the recruited patients. The patients and controls were divided into two cohorts, cohort WES and cohort WGS.

The cohort WES included PD patients with AAO no more than 50 years or with a family history of PD, and control participants, who were assessed using WES. One hundred and twenty-one patients with pathogenic/likely pathogenic variants of high- or very high-confidence PD disease-causing genes [22] were excluded from the cohort and the related study was accepted and in press. A large sample of sporadic late-onset (AAO > 50) PD patients, and the age/sex-matched healthy controls were included in the cohort WGS.

All participants provided written informed consent for participation in the genetic research, which was approved by relevant oversight committees and institutional review boards. Genomic DNA was prepared from peripheral blood leukocytes according to standard procedures. Of note, 13.8% (973/7030) of the participants overlapped with those in our previous study [10] of rare *GCH1* variants in PD patients analyzed by molecular inversion probe technique, including 891 cases and 82 controls.

A comprehensive dataset of basic demographic data including age, sex, family history, disease duration and clinical features including motor and non-motor manifestations, was collected from the recruited PD patients, and saved into the PD-MDCNC database.

### Genotyping and quality control

WES and WGS were performed according to previous descriptions [23, 24]. Burrows-Wheeler Aligner-MEM algorithm [25], Picard (http://broadinstitute.github.io/picard/), and Genome Analysis Toolkit [26] were used to generate high-quality variants. ANNOVAR [27] and VarCards [28] were used to annotate the variants with gene regions (RefSeq, hg19), amino acid alterations, functional effects, and allele frequencies in East Asian population (gnomAD database, ExAC database). And ReVe [29] was used for in silico pathogenicity prediction (threshold, 0.7).

For individual and variant quality control, the PLINK software v1.90 was used [30]. Individuals were excluded if they showed ambiguous sex (conflicting sex assignment in PLINK), low genotype call rates (missing rate > 10%), deviating heterozygosity/genotype calls (± 3 standard deviations [SDs]), or cryptic relatedness (identity by descent > 0.15). Variant quality control was accomplished by removal of low-quality genotypes (Phred-scaled genotype quality score below 30, allele depth [AD] below 10, and reads depth [DP] below 30 for WES data; Phred-scaled genotype quality score below 15, AD below 2, and DP below 5 for WGS data) and variants with low call rates (missing rate > 10%) or departure from Hardy-Weinberg equilibrium (*P* < 0.0001). Principal component analysis for population stratification was conducted using independent high-quality variants and main principal component variables for each sample were obtained. Outliers (suggesting non-Chinese ancestry) were excluded from further analysis.

### Selection of *GCH1* variants

The *GCH1* transcript region including UTRs, exons and introns (NM_000161 at chr14:55,308,724-55,369,542; hg19) [31], regulatory region from Genehancer [32], brain-specific eQTLs from Genotype-Tissue Expression (GTEx) project [33], and significant GWAS signals [12] were considered in the study (Table S1), and the corresponding quality parameters of the targeted *GCH1* regions are presented in Table S2. The variants from target *GCH1* regions were extracted and divided into coding variants or non-coding variants as per their position in the genome, and common or rare variants on the basis of the minor allele frequencies (MAF) in covered samples of cohorts WES and WGS at a threshold of 0.01 (MAF < 0.01 into rare variants and others into common variants). The coding variants were further categorized into missense, synonymous, loss-of-function (stop gain/loss, frameshift, and splicing mutations falling within two base pairs of exon-intron junctions), protein-altering (missense and loss-of-function), and deleterious (predicted to be damaging by ReVe or previously reported to be associated with PD or DRD). Specifically, the Gene4PD database (http://www.genemed.tech/gene4pd/) developed by our group, the MDSgene database (https://www.mdsgene.org/), and PubMed were searched to determine if these variants had been reported [8, 10, 34–38] to be associated with PD or DRD. The non-coding variants were assorted into UTRs/introns, eQTLs, GWAS signals, and regulatory elements according to the genomic location and functional property.

### Meta-analysis

Literature was searched in electronic databases including PubMed, Embase and Cochrane Library using a combination of following keywords: “GCH1”, “guanosine triphosphate cyclohydrolase 1”, “GTP cyclohydrolase 1”, “Parkinson disease”, “Parkinson’s disease”, and “Parkinsonism”. The last search was updated on April 1, 2020. Two researchers performed the search independently and in case of a disagreement, a third researcher was consulted to arrive at a consensus.

Eligible studies were included according to the following criteria: (1) should be an observational study, such as using a case-control or cohort design; (2) all PD patients should be diagnosed according to the UK brain bank criteria [20] or MDS clinical diagnostic criteria for PD [21]; and (3) clearly reporting results of deleterious *GCH1* variants and corresponding AAO data. Moreover, the following studies were excluded: (1) case reports, editorials, reviews or functional research; (2) duplicate studies (when multiple studies employed the same participants, the latest or most complete report was included); and (3) studies with incomplete data, which did not specify the AAO among carriers of *GCH1* deleterious variants.

Data were extracted for meta-analysis by two researchers independently, including first author’s name, year of publications, ethnicity and country of participants, gene and variants, numbers of PD patients with and without *GCH1* deleterious variants, and their corresponding AAO information. Any disagreement was resolved by the senior authors. The quality of the included studies was evaluated by the Newcastle-Ottawa Scale [39], which came out to be approximately good for the present study.

### Statistical analysis

For common variant association analysis, PLINK [30] was used to perform logistic regression with age, sex and first five principal components for population stratification as covariates. To adjust for multiple comparisons, Bonferroni-corrected significance threshold was used to determine the statistical difference. We applied spectral decomposition adjustment using the SNPSpD method (https://gump.qimr.edu.au/general/daleN/SNPSpD) to determine the number of independent statistical tests in over 200 common variants. The source of each common variant is shown in Table S3. The estimated number of independent tests was 19, corresponding to a Bonferroni-corrected significance threshold of *P* < 0.003.

For variant set analysis, the sequence kernel association test (SKAT) implemented by the SKAT R package was used. The optimized SKAT (SKAT-O) was used to analyze the rare variant association in a specified region and combined SKAT (SKAT-C) was used to evaluate the cumulative effect of all variants including both rare and common variants on disease risk [40, 41]. Covariates were included to adjust the analyses for sex, age and first five principal components of ancestry. The estimated number of independent tests was 11, corresponding to a Bonferroni-corrected significance threshold of *P* < 0.0045. We considered those results with a *P*-value < 0.05, but not surviving the Bonferroni correction, as ‘suggestive’.

For genotype-phenotype correlation assessment, linear and logistic regression analyses were performed using PLINK, with adjustment for age at entry, disease duration, and sex. The estimated number of independent tests was 22, corresponding to a Bonferroni-corrected significance threshold of *P* < 0.0023.

Meta-analysis of the association of deleterious variants of *GCH1* with AAO of PD patients was carried out, which included our own data from this study. To assess the importance of *GCH1* deleterious variants on the AAO of PD patients, continuous data were expressed as pooled mean difference with 95% confidence intervals (CI). The heterogeneity across studies was evaluated by Higgin’s *I*^2^ index. If the heterogeneity (*P* > 0.10, *I*^2^ < 50%) was not significant, the fixed effect model was adopted, otherwise the random effect model was used. The reporting biases were assessed by funnel-plot analysis. All analyses were done using the Review Manager software package v.5.3 (The Cochrane Collaboration, Oxford, England).

## Results

### Cohort description

The WES cohort included 1555 PD patients with AAO ≤ 50 years or with a family history, and 2234 controls without any neurological disease. The PD patients had a mean age of 52.3 ± 8.9 years and a mean AAO of 46.0 ± 8.3 years; 54.3% (845/1555) of them were male and 23.0% (358/1555) reported a positive family history. The mean age of control participants was 42.8 ± 8.7 years and 60.2% (1345/2234) were male. The average sequencing depth in the WES cohort was 123-fold and 99.32% of targeted regions achieved a minimum of 10 coverage.

The WGS cohort consisted of 1962 PD patients with sporadic late-onset PD (AAO > 50 years) and 1279 age/sex-matched controls. The PD cases were recruited at a mean age of 66.8 ± 7.1 years and had a mean AAO of 61.9 ± 6.9 years, and 50.2% (984/1962) were male. Control participants had an average age of 59.3 ± 7.1 years and 48.0% (613/1279) were male. The average sequencing depth in the WGS cohort was 12-fold and 95.5% of genome regions were covered at least 5-fold.

**Fig. 1:**
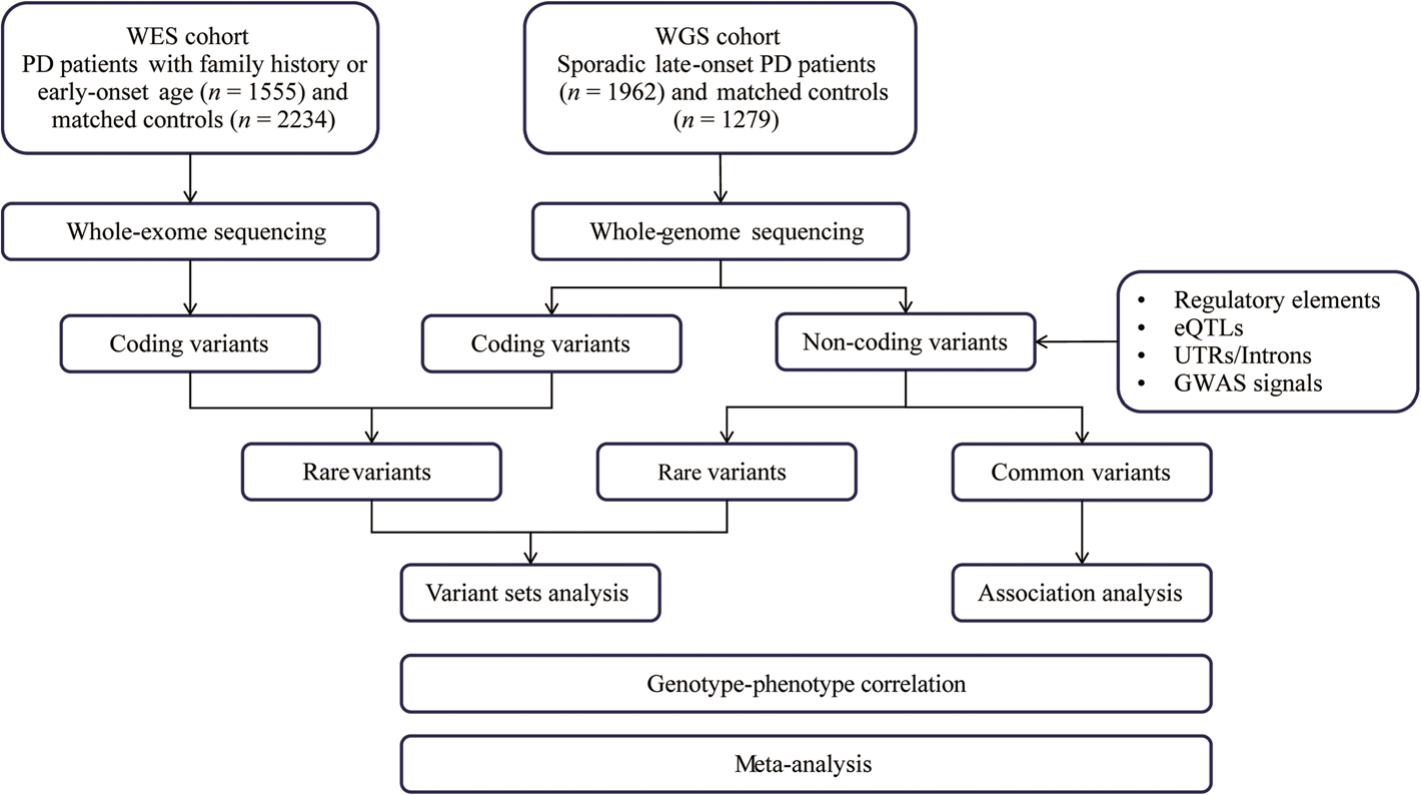
Workflow of this study

The workflow of the study is presented in Fig. 1. The detailed information about the target variants and regions related to *GCH1* included in the study are shown in Table S1. The baseline characteristics of the demographic data, the motor and non-motor symptoms of patients from cohort WES and cohort WGS are presented in Table S4.

### Coding variants

Both cohorts WES and WGS were subjected to coding variant analysis, which included six exons of the coding variants. Of note, all the identified coding variants were rare (Table S5).

**Fig. 2:**
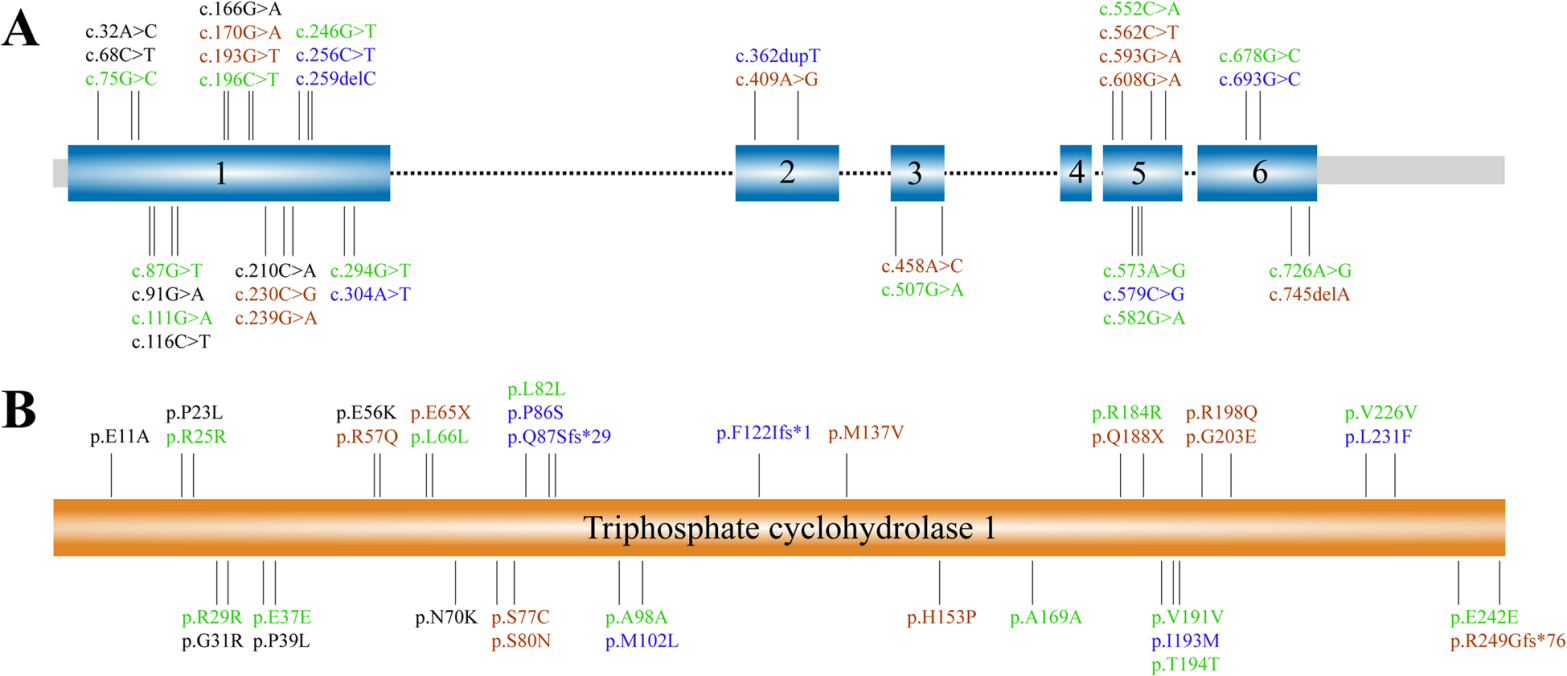
Schematics of *GCH1* and its triphosphate cyclohydrolase 1. Synonymous variants (green), previously reported variants associated with PD or DRD (red), newly described variants with predicted damaging functional alterations (blue) and variants with predicted tolerable functional alterations (black)

In the WES cohort, a significant association of *GCH1* coding variants with PD was found (1.6% vs 0.5%, SKAT-O, *P* = 0.0001). Further stratification by variant properties showed that the association mainly involved the protein-altering variants (1.2% vs 0.2%, SKAT-O, *P* < 0.0001). Interestingly, 19 variants were predicted to be damaging or had been previously reported in PD or DRD patients (defined as “deleterious”, Fig. 2), of which significant associations were detected (1.2% vs 0.1%, SKAT-O, *P* < 0.0001) in the WES cohort (Table 1). In the cohort WGS, however, no association was observed for either all variants (1.0% vs 0.9%, SKAT-O, *P* = 0.70) or deleterious variants (0.5% vs 0.2%, SKAT-O, *P* = 0.30) (Table 1).

**Table 1:** Burden analysis of *GCH1* coding variants

Variant type	WES cohort			WGS cohort		
	Case (*n* = 1555)	Control (*n* = 2234)	*P* value	Case (*n* = 1962)	Control (*n* = 1279)	*P* value
All	25	11	0.0001	19	12	0.70
Synonymous	6	6	0.16	7	5	0.93
Loss of function	5	0	0.008	0	0	> 0.99
Missense	14	5	0.0009	12	7	0.55
Protein-altering	19	5	< 0.0001	12	7	0.55
Deleterious	19	3	< 0.0001	10	3	0.33

Deleterious variants are predicted to be damaging or have previously been reported to be associated with PD or DRD. Loss of function indicates stop gain/loss, frameshift, and splicing mutations falling within two base pairs of exon-intron junctions. The protein-altering variants include missense and loss-of-function variants. The *P* values were analyzed by SKAT-O

### Non-coding variants

Non-coding variant analysis was only conducted in the WGS cohort and a total of 1702 variants (including those in the regulatory elements, eQTLs, UTRs/introns and GWAS signals, respective numbers of which are shown in Table S1) were identified, including 1486 rare variants and 216 common variants.

**Fig. 3:**
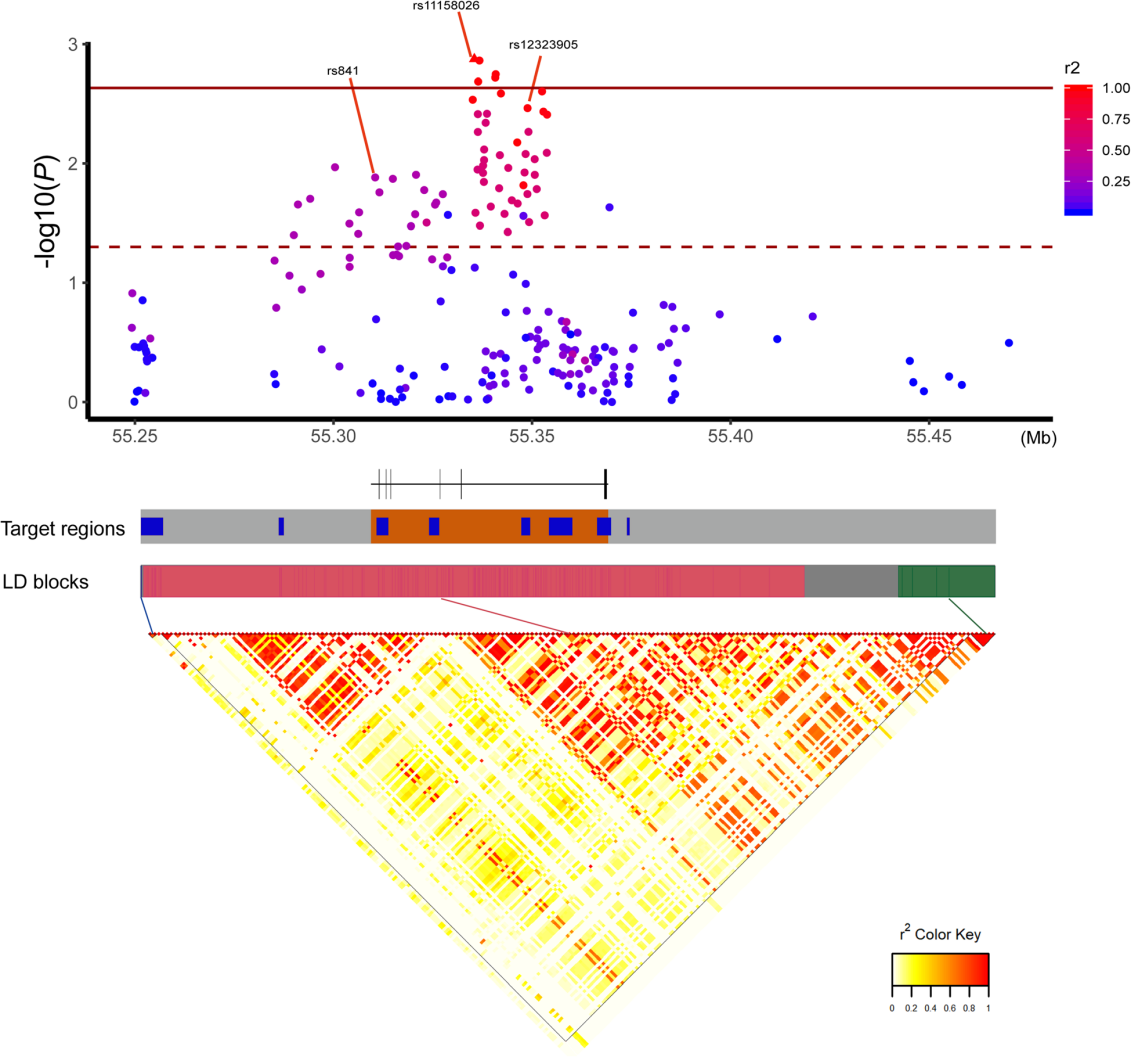
Target *GCH1* regions with associative results and linkage disequilibrium. The top scatter plot shows the association analysis of *P*-values in the WGS cohort and degree of linkage disequilibrium (LD) with the lead single variant. The solid line marks the Bonferroni-corrected significance threshold and the dashed line marks the suggestive significance threshold of *P* < 0.05. In the target regions, gray color depicts the range of eQTLs, blue color depicts regulatory regions, and orange shows transcript region. The bottom plot demonstrates the LD blocks of target regions. *Three variants were not included in the figure because these were in the distal region of present targets and were not associated with PD.

Among the 216 common variants (Table S3), we identified that rs12323905 remained significantly associated with PD after multiple comparison correction in our data (odds ratio = 1.19, 95%CI 1.07–1.32, *P* = 0.001). The previously reported associated variants rs841 (odds ratio = 0.87, 95%CI 0.78–0.97, *P* = 0.01) and GWAS signal rs11158026 (odds ratio = 1.17, 95%CI 1.06–1.3, *P* = 0.003) were also found to be associated with PD in the same association direction [9, 12], however, these associations did not remain significant after multiple comparison correction. Of note, both rs11158026 (*r*^2^ = 0.98) and rs841 (*r*^2^ = 0.34) were linked to the top significant variant rs12323905 in our dataset (Fig. 3).

To determine which sets of *GCH1* non-coding variants were associated with the PD risk, we performed exploratory analyses using SKAT-O and SKAT-C to assess the potential contribution of variants within different sets considered independently. In the collapse analysis of rare variants, variants from both UTR/intron regions (SKAT-O, *P* = 0.80) and eQTLs (SKAT-O, *P* = 0.18) did not show any significant association with PD, whereas one of the promoter enhancer sets demonstrated suggestive difference between PD patients and controls (GH14J054857, SKAT-O, *P* = 0.03). Moreover, suggestive differences were observed in combinations of common and rare variants from each variant set. The UTR/intron regions (SKAT-C, *P* = 0.03), eQTLs (SKAT-C, *P* = 0.02) and a regulatory region (GH14J054880, SKAT-C, *P* = 0.02) were detected with significant differences (Table 2). However, none of them remained significant after multiple comparison correction.

**Table 2:** Burden analysis of *GCH1* non-coding variants in the WGS cohort

Groups	Items	Rare variant set analysis		All variant set analysis	
		Variants included	*P* value (SKAT-O)	Variants included	*P* value (SKAT-C)
UTRs/introns (NM_000161)	UTRs/introns	1258	0.80	1403	0.03
Promoter/Enhancer	GH14J054782	128	0.44	146	0.41
	GH14J054818	24	1	28	0.50
	GH14J054843	58	0.46	63	0.09
	GH14J054857	44	0.03	47	0.05
	GH14J054880	35	0.28	50	0.02
	GH14J054889	134	0.74	149	0.82
	GH14J054900	79	0.41	90	0.37
	GH14J054908	9	0.73	11	0.35
	All	511	0.72	584	0.15
eQTLs	brain specific eQTLs	7	0.18	119	0.02

### Genotype-phenotype correlation

Next, we analyzed the phenotypic characteristics of *GCH1* variant-carriers. We found that patients with deleterious variants had younger AAO than non-carriers in the WES cohort (*P* < 0.0001, Table 3), and the suggestive association was even observed in the WGS cohort (*P* = 0.048, Table 4) where the burden of deleterious variants did not significantly differ from the controls. No significant association was found between rs12323905 and AAO in the WGS cohort. In addition, patients with deleterious variants in the WES cohort showed milder motor symptoms (UPDRS II, *P* = 0.022; UPDRS III, *P* = 0.025), reduced bradykinesia symptoms (*P* = 0.024), and less severe fatigue scale (*P* = 0.035) with suggestive significance (Table 3), which suggested that deleterious variants were not only related to the risk of PD, but also suggestively associated with the alleviated motor symptoms. Likewise, in the WGS cohort, we observed suggestive association between deleterious variants and more autonomic nervous dysfunction, as well as between rs12323905 and lesser fatigue symptoms.

**Table 3:** Comparison of clinical features in carriers and non-carriers of deleterious *GCH1* variants in the WES cohort

Clinical features	Deleterious variants			
	Carriers	Non − carriers	β / OR	*P* value
Age at onset (years) ^a^	38.89 ± 16.44	46.12 ± 8.07	−7.97	< 0.0001
Age at assessment (years) ^a^	44.50 ± 150	52.38 ± 8.79	−7.96	< 0.0001
Disease duration (years) ^b^	5.61 ± 6.30	6.26 ± 5.16	1.36	0.21
UPDRS−Part I	1.63 ± 1.74	2.45 ± 2.08	−0.63	0.18
UPDRS−Part II	7.26 ± 4.79	12.03 ± 6.87	−3.23	0.02
UPDRS−Part III	16.58 ± 12.37	27.63 ± 15.97	−7.69	0.03
Tremor score	2.63 ± 3.72	3.80 ± 3.77	−0.69	0.42
Stiffness score	3.26 ± 3.33	5.64 ± 4.25	−1.7	0.08
Bradykinesia score	5.90 ± 5.03	10.28 ± 6.67	−3.31	0.02
Postural instability score	2.47 ± 2.32	4.08 ± 3.20	−0.98	0.14
Hoeh and Yahr stage	1.79 ± 0.82	2.20 ± 0.85	−0.30	0.09
Dyskinesia	5.56%	16.44%	0.13	0.07
Freezing gait	11.11%	27.64%	0.33	0.15
Motor subtype				
Tremor-dominant	26.32%	26.56%	–	–
Intermediate	5.26%	17.51%	0.28	0.25
PIGD−dominant	68.42%	55.92%	1.29	0.64
MMSE	28.58 ± 1.56	27.01 ± 3.20	1.49	0.09
PDSS	116.50 ± 30.89	115.80 ± 29.67	−2.85	0.73
RBDQ−HK	13.67 ± 16.60	13.39 ± 16.01	2.64	0.56
ESS	8.27 ± 6.29	7.37 ± 6.08	1.92	0.28
HAMD	2.67 ± 2.23	5.89 ± 5.62	−0.27	0.88
HRS	20.42 ± 5.63	19.89 ± 6.37	−2.92	0.07
PFS	39.89 ± 16.15	44.22 ± 18.98	−14.62	0.04
SCOPA−AUT	5.30 ± 7.65	7.40 ± 6.87	−1.70	0.79

Results are from linear or logistic regression analyses adjusting for age, sex, BMI, and ancestry. *UPDRS* Unified Parkinson’s disease rating scale, *MMSE* Mini-mental state examination, *PDSS* Parkinson’s disease sleep scale, *RBDQ-HK* Rapid eyes movement sleep behavior disorder questionnaire-Hong Kong, *ESS* Epworth sleepiness scale, *HAMD* 17-item Hamilton depression rating Scale, *HRS* Hyposmia rating Scale, *PFS* Parkinson’s disease fatigue scale, *SCOPA-AUT* Scales for outcomes in Parkinson’s disease-autonomic, *PDQ39* The 39-item Parkinson’s disease Questionnaire, *PFS* Parkinson’s disease fatigue scale. According to the score from UPDRS, motor subtype [42] was classified as tremor-dominant (TD) phenotype when the ratio of tremor score to postural instability and gait difficulty (PIGD) score was no less than 1.5, whereas patients with a ratio of no more than 1.0 were defined with PIGD phenotype, and the rest of patients belonged to the indeterminate phenotype

^a^Adjusted for sex and disease duration at entry

^b^Adjusted for sex and age at entry

**Table 4:** Comparison of clinical features in carriers and non-carriers of different *GCH1* variants in the WGS cohort

Clinical features	Deleterious variants				rs12323905			
	Carriers	Non − carriers	β / OR	*P* value	Carriers	Non − carriers	β / OR	*P* value
Age at onset (years) ^a^	58.90 ± 3.70	61.90 ± 6.94	−4.24	0.048	61.81 ± 6.94	61.89 ± 6.79	0.07	0.76
Age at assessment (years) ^a^	61.25 ± 3.90	66.85 ± 7.07	−4.24	0.048	66.77 ± 7.10	66.66 ± 6.87	0.07	0.76
Disease duration (years) ^b^	2.35 ± 1.42	4.92 ± 3.60	−1.76	0.11	4.93 ± 3.70	4.73 ± 3.32	0.14	0.25
UPDRS−Part I	3.00 ± 2.05	2.60 ± 2.08	0.61	0.35	2.58 ± 2.07	2.57 ± 1.95	0.02	0.72
UPDRS−Part II	12.30 ± 6.98	12.34 ± 6.37	2.13	0.25	12.23 ± 6.35	12.27 ± 6.24	−0.05	0.80
UPDRS−Part III	26.4 ± 19.07	27.76 ± 14.04	2.08	0.62	27.53 ± 14.04	27.90 ± 13.77	0.02	0.96
Tremor score	2.90 ± 2.51	3.45 ± 3.43	−0.13	0.90	3.40 ± 3.37	3.59 ± 3.44	−0.08	0.47
Stiffness score	5.80 ± 5.05	5.57 ± 4.12	0.53	0.68	5.48 ± 4.13	5.70 ± 4.06	−0.02	0.87
Bradykinesia score	10.00 ± 7.38	10.22 ± 6.11	0.92	0.63	10.12 ± 6.09	10.33 ± 5.95	0.03	0.88
Postural instability score	4.40 ± 3.75	4.51 ± 2.93	1.03	0.22	4.54 ± 2.94	4.31 ± 2.88	0.10	0.25
Hoeh and Yahr stage	2.00 ± 0.82	2.03 ± 0.74	0.24	0.27	2.03 ± 0.74	2.01 ± 0.75	0.02	0.46
Dyskinesia	0.00%	10.00%	–	–	9.01%	9.05%	0.96	0.77
Freezing gait	10.00%	23.74%	0.58	0.61	21.92%	20.21%	1.02	0.84
Motor subtype								
Tremor-dominant	10.00%	21.77%	–	–	21.18%	22.67%	–	–
Intermediate	30.00%	15.97%	4.16	0.22	15.59%	19.33%	0.89	0.32
PIGD−dominant	60.00%	62.27%	2.76	0.35	63.23%	58.00%	1.05	0.61
MMSE	26.80 ± 3.52	25.56 ± 4.40	0.29	0.83	25.54 ± 4.37	25.77 ± 4.22	−0.11	0.44
PDSS	107.8 ± 21.35	112.40 ± 29.21	−10.02	0.27	113.3 ± 28.36	110.70 ± 30.45	1.58	0.10
RBDQ−HK	9.60 ± 11.76	16.07 ± 17.31	−3.69	0.50	15.94 ± 17.13	16.69 ± 18.17	−0.52	0.38
ESS	7.90 ± 4.75	8.42 ± 6.50	0.08	0.97	8.33 ± 6.49	8.43 ± 6.50	−0.03	0.90
HAMD	8.40 ± 5.97	6.03 ± 5.47	0.51	0.80	6.04 ± 5.54	6.11 ± 5.20	−0.04	0.84
HRS	19.60 ± 4.77	18.71 ± 6.93	13.27	0.08	18.62 ± 7.01	19.02 ± 6.717	−0.32	0.17
PFS	51.33 ± 18.53	46.46 ± 19.13	8.42	0.28	45.89 ± 19.14	47.89 ± 19.32	−1.72	0.03
SCOPA−AUT	13.43 ± 8.46	9.44 ± 7.22	6.11	0.02	9.54 ± 7.39	8.97 ± 6.71	0.20	0.46

Results are from linear or logistic regression analyses adjusting for age, sex, BMI, and ancestry

*UPDRS* Unified Parkinson’s disease rating scale, *MMSE* Mini-mental state examination, *PDSS* Parkinson’s disease sleep scale, *RBDQ-HK* Rapid eyes movement sleep behavior disorder questionnaire-Hong Kong, *ESS* Epworth sleepiness scale, *HAMD* 17-item Hamilton depression rating Scale, *HRS* Hyposmia rating Scale, *SCOPA-AUT* Scales for outcomes in Parkinson’s disease-autonomic, *PDQ39* The 39-item Parkinson’s disease Questionnaire, *PFS* Parkinson’s disease fatigue scale

^a^Adjusted for sex and disease duration at entry

^b^Adjusted for sex and age at entry

### Meta-analysis of *GCH1* deleterious variants and AAO

**Fig. 4:**
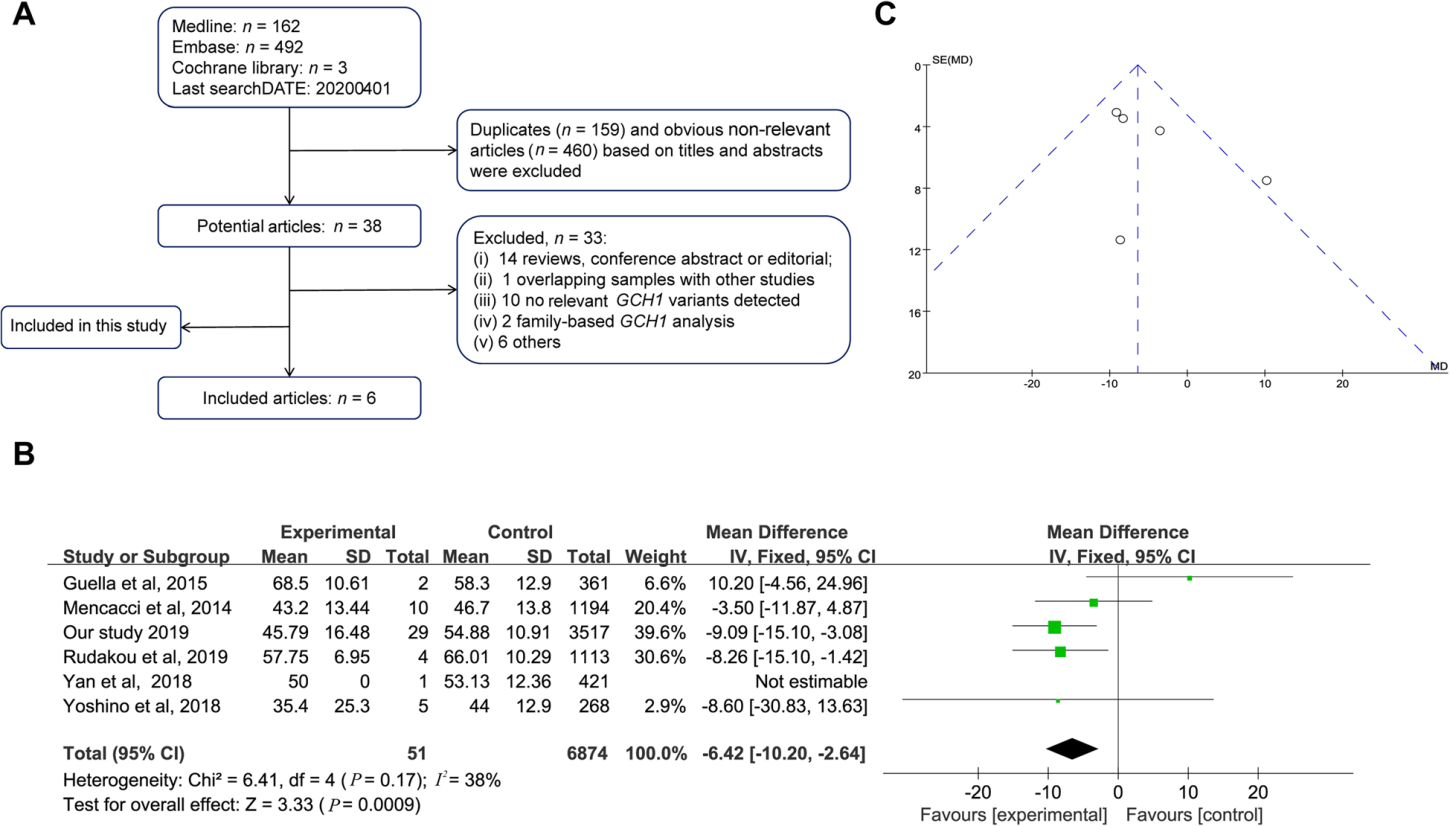
Meta-analysis of *GCH1* deleterious variants and AAO. **a** Flowchart showing the search strategy and selection criteria of literature for meta-analysis; **b** Forest plot of AAO of PD in *GCH1* deleterious variant-carriers and non-carriers; **c** Funnel plot of AAO of PD in *GCH1* deleterious variant-carriers and non-carriers

The workflow of the meta-analysis is shown in Fig. 4a. A total of 51 *GCH1* deleterious variant-carriers and 6874 PD patients from six studies (including the present study) were included to analyze the association between *GCH1* status and AAO in PD patients. The characteristics of the included studies [8, 9, 14, 43, 44] are shown in Fig. 4b. The funnel plot obtained was showed in Fig. 4c. The AAO in *GCH1* deleterious variant-carriers was more than 6 years earlier than non-carriers [MD: − 6.42 (− 10.20, − 2.64); *P* = 0.0009; Fig. 4b]. Of note, meta-analysis conducted after exclusion of our study data showed borderline significance [MD: − 4.67 (− 9.53, 0.20); *P* = 0.06].

## Discussion

Over the past decades, investigations of coding and non-coding genome in PD have identified over 20 disease-causing genes and about 90 risk loci in PD [12, 22, 45]. However, most of them were identified in European ancestry populations, while little is known about the genetics of PD in other populations. With the decreased sequencing cost and development of new high-throughput technologies, systematic genetic studies in other populations have become possible. In this study, we identified that both *GCH1* coding and non-coding variants are involved in PD. We found that the deleterious variants could not only increase the risk of PD, but also advance the AAO of PD by over 6 years. We also identified that rs12323905, UTRs/introns, eQTLs, and two regulatory regions may be associated with PD and the *GCH1* deleterious variants may influence PD manifestations.

A total of 19 and 12 protein-altering variants of *GCH1* were detected in cohorts WES and WGS, respectively. Apart from the earlier reported variants, we also found several novel missense or loss-of-function variants of *GCH1* in our PD cohort, including p.I193M (*n* = 2), p.F122Ifs*1, p.M102L, p.Q87Sfs*29, p.P86S, p.P39L, and p.E11A (*n* = 1 each), of which p.I193M, p.F122Ifs*1, p.Q87Sfs*29, and p.P86S were predicted to be deleterious in our in silico analysis (Table S5). Increased burden of deleterious variants in *GCH1* gene was detected in PD patients of the WES cohort, but not in the late-onset patients of the WGS cohort, which was in line with previous studies on late-onset PD [13], suggesting that the pathophysiology of PD with early-onset or familial history may differ from that of the late-onset PD. This is also supported by the observation that the incidence and prevalence of late-onset PD rapidly increased with age, whereas the incidence of early-onset PD is low [46].

To our knowledge, this is the first study to systematically analyze the association between *GCH1* non-coding variants and PD risk using different sequencing techniques, especially WGS. Of note, the unreported rs12323905 was found to be significantly associated with PD in Chinese population. To date, the most significant variant in *GCH1* locus revealed by GWAS in European populations was rs11158026 [11, 12]. The difference in top significant variants between different populations indicates the genetic heterogeneity among PD populations; and combination of results from different populations could provide insights into the mapping of true causal variants in *GCH1* locus. Overall, our study confirmed that the GWAS signal reported in European populations has significance for Chinese populations as well, but there is a difference in the top significant variant, which warrants further functional studies to determine these associations in PD. Furthermore, our study revealed that variants in GH14J054857, GH14J054880, UTRs/introns and brain-specific eQTLs sets of *GCH1* may play a role in PD pathogenesis, especially the rare variants in GH14J054857 sets. The suggestive correlations still need to be further replicated and explored. Meanwhile, the suggestive results of *GCH1* non-coding variants may highlight a possible link between long-lasting alterations in dopamine synthesis and an increased risk of PD development [6]. Further mechanistic studies may provide insights into the basis of common diseases along with a potential explanation for how *GCH1* correlates with the risk of PD development.

In the WES cohort, genotype-phenotype analysis showed that the carriers of deleterious variants had younger AAO, which indicates the role of *GCH1* in the increased risk of PD as well as in altered AAO in PD patients. However, there was no significant difference between common variants of *GCH1* and AAO, which was in line with a recent largest AAO GWAS study [47]. In addition, we did a meta-analysis of 6874 PD patients including 51 *GCH1* carriers from 6 studies to evaluate the potential association between the *GCH1* deleterious variants and AAO of the patients, which demonstrated that the *GCH1* deleterious variants could modify the AAO in PD patients (more than 6 years ahead). Due to the limited number of PD patients carrying *GCH1* deleterious variants, the association of the *GCH1* deleterious variants with AAO only showed broadline significance, when the current study was not included. Furthermore, these are some preliminary suggestive findings between *GCH1* variants and clinical manifestations in PD such as milder motor symptoms and more autonomic nervous dysfunction in deleterious variant-carriers. Several studies [8, 43] simply described the specific symptoms of PD patients carrying each *GCH1* variant without a quantifying scale and comparisons. In our study, we used a specific rating scale to quantify the symptom severity and systematically compared the phenotypic difference between *GCH1* variant-carriers and non-carriers. Given that the suggestive associations were not significant after Bonferroni correction and might be biased due to the insufficient number of *GCH1* variant-carriers, these findings need further explorations in future.

There were several limitations in this study. First, the copy number variations encompassing the *GCH1* locus were not under the scope of the analysis. Second, we did not explore the association of non-coding variants in subjects from cohort WES, given that the WES method could not fully detect the non-coding variants. Third, further studies to identify the functional mechanisms are warranted, including how these associated factors affect the functional roles of *GCH1* and how changes in *GCH1* can lead to PD pathogenesis. Fourth, studies focused on multiple aspects such as epistasis and environmental factors are also needed. Fifth, the late-onset PD patients were detected with low-coverage WGS; although we have filtered the variants through reasonable quality control, it is still worth further replication in other cohorts.

## Conclusions

In conclusion, deleterious variants of *GCH1* and non-coding signals especially rs12323905 were found to be associated with PD in Chinese mainland population. Clinically, rare or common variants of *GCH1* may modify the phenotype of PD patients in motor and non-motor aspects, respectively. Meta-analysis further demonstrated that the rare deleterious variants of *GCH1* could modify the AAO of PD. Our results highlight the importance of *GCH1* locus in the risk of PD development as well as in the AAO of PD, and provide reference for experimental study design to elucidate the mechanisms of *GCH1* involvement in the pathogenesis of PD.

## Supplementary information

**Additional file 1 Table S1.** Targeted *GCH1* regions and variants included in the study.

**Additional file 2 Table S2.** The quality control of the targeted *GCH1* regions.

**Additional file 3 Table S3.** Association analysis of common non-coding variants identified in this study.

**Additional file 4 Table S4.** Summary of clinical features of the Parkinson’s disease patients in this study.

**Additional file 5 Table S5.** Coding variants of *GCH1* identified in two cohorts.

## Data Availability

The datasets supporting the conclusions of this article are included within the article and its additional files.

## Abbreviations

PD: Parkinson’s disease
DRD: Dopa-responsive dystonia
*GCH1*: Guanosine triphosphate cyclohydrolase 1
GWAS: Genome-wide association studies
eQTLs: Expression quantitative trait loci
AAO: Age at onset
WES: Whole-exome sequencing
WGS: Whole-genome sequencing
UTRs: Untranslated regions
PD-MDCNC: Parkinson’s Disease & Movement Disorders Multicenter Database and Collaborative Network in China
MDS: Movement Disorders Society
GTEx: Genotype-Tissue Expression
MAF: Minor allele frequencies
SKAT: Sequence kernel association test
MD: Mean difference
CI: Confidence intervals
SD: Standard deviation
