# Supplementary material for: GCH1 variants contribute to the risk and earlier age-at-onset of Parkinson’s disease: a two-cohort case-control study
Source: Transl Neurodegener. 2020 Aug 4;9:31. doi: 10.1186/s40035-020-00212-3 (PMC7401216; doi:10.1186/s40035-020-00212-3)
Supplement: Supplementary file 1 — Additional file 1 Table S1. Targeted GCH1 regions and variants included in the study. [file 40035_2020_212_MOESM1_ESM.docx]

**Table S1. Targeted *GCH1* regions and variants included in the study**

| Groups | Items | Position (hg19) | Variants identified | Common variants ^a^ | Rare variants ^a^ | Cohort analyzed | Source |
| --- | --- | --- | --- | --- | --- | --- | --- |
| Coding region (NM_000161) | Exon1 | chr14:55369037-55369381 | 19 | 0 | 19 | Cohort WES+WGS | UCSC |
|  | Exon2 | chr14:55332043-55332156 | 2 | 0 | 2 | Cohort WES+WGS | UCSC |
|  | Exon3 | chr14:55326397-55326456 | 2 | 0 | 2 | Cohort WES+WGS | UCSC |
|  | Exon4 | chr14:55313815-55313850 | 0 | 0 | 0 | Cohort WES+WGS | UCSC |
|  | Exon5 | chr14:55312484-55312572 | 7 | 0 | 7 | Cohort WES+WGS | UCSC |
|  | Exon6 | chr14:55310735-55310863 | 4 | 0 | 4 | Cohort WES+WGS | UCSC |
| UTRs/introns  (NM_000161) | 5’UTR | chr14:55369382-55369544 | 4 | 1 | 3 | Cohort WGS | UCSC |
|  | Intron1 | chr14:55332157-55369036 | 884 | 106 | 778 | Cohort WGS | UCSC |
|  | Intron2 | chr14:55326457-55332042 | 119 | 11 | 108 | Cohort WGS | UCSC |
|  | Intron3 | chr14:55313851-55326396 | 275 | 21 | 254 | Cohort WGS | UCSC |
|  | Intron4 | chr14:55312573-55313814 | 35 | 0 | 35 | Cohort WGS | UCSC |
|  | Intron5 | chr14:55310864-55312483 | 38 | 3 | 35 | Cohort WGS | UCSC |
|  | 3’UTR | chr14:55308735-55310734 | 48 | 3 | 45 | Cohort WGS | UCSC |
| Promoter/  Enhancer | GH14J054782 | chr14:55367131-55370718 | 146 | 18 | 128 | Cohort WGS | GeneHancer |
|  | GH14J054818 | chr14:55354720-55360717 | 28 | 4 | 24 | Cohort WGS | GeneHancer |
|  | GH14J054843 | chr14:55284720-55286138 | 63 | 5 | 58 | Cohort WGS | GeneHancer |
|  | GH14J054857 | chr14:55323720-55326317 | 47 | 3 | 44 | Cohort WGS | GeneHancer |
|  | GH14J054880 | chr14:55347587-55349794 | 50 | 15 | 35 | Cohort WGS | GeneHancer |
|  | GH14J054889 | chr14:55374820-55375489 | 149 | 15 | 134 | Cohort WGS | GeneHancer |
|  | GH14J054900 | chr14:55249195-55254917 | 90 | 11 | 79 | Cohort WGS | GeneHancer |
|  | GH14J054908 | chr14:55310086-55313118 | 11 | 2 | 9 | Cohort WGS | GeneHancer |
| eQTLs | 145 brain specific eQTLs | chr14:55183314-56227190 | 119 | 112 | 7 | Cohort WGS | GTEx |
| GWAS signals | rs11158026 | chr14:55348869 | 1 | 1 | 0 | Cohort WGS | Published literature |
| Total ^b^ |  |  | 1702 | 216 | 1486 |  |  |

UCSC = University of California, Santa Cruz Genome Browser; GTEx = The Genotype-Tissue Expression project.

a. The common or rare variants were defined by the minor allele frequencies in covered samples of cohort WES and WGS at the threshold of 0.01.

b. The total does not equal the sum of the listed items because different groups of target region may overlap
