# Supplementary material for: GCH1 variants contribute to the risk and earlier age-at-onset of Parkinson’s disease: a two-cohort case-control study
Source: Transl Neurodegener. 2020 Aug 4;9:31. doi: 10.1186/s40035-020-00212-3 (PMC7401216; doi:10.1186/s40035-020-00212-3)
Supplement: Supplementary file 2 — Additional file 2 Table S2. The quality control of the targeted GCH1 regions. [file 40035_2020_212_MOESM2_ESM.docx]

**Table S2. The quality control of the targeted *GCH1* regions**

| **Items** | **Targeted regions in WES** | **Targeted regions in WGS** |
| --- | --- | --- |
|  | **Mean ± SD** | **Mean ± SD** |
| Mean depth | 80.54±16.98 | 11.57±1.22 |
| Coverage ≥ 1× (%) | 99.83±1.15 | 99.99±0.01 |
| Coverage ≥ 5× (%) | 99.33±2.68 | 99.44±0.42 |
| Coverage ≥ 10× (%) | 98.01±5.33 | 75.10±9.37 |
| Coverage ≥ 30× (%) | 93.58±11.84 | - |
