# Supplementary material for: GCH1 variants contribute to the risk and earlier age-at-onset of Parkinson’s disease: a two-cohort case-control study
Source: Transl Neurodegener. 2020 Aug 4;9:31. doi: 10.1186/s40035-020-00212-3 (PMC7401216; doi:10.1186/s40035-020-00212-3)
Supplement: Supplementary file 3 — Additional file 3 Table S3. Association analysis of common non-coding variants identified in this study. [file 40035_2020_212_MOESM3_ESM.docx]

**Table S3. Association analysis of common non-coding variants identified in this study**

| **Position (hg19)** | **SNP ID** | **Minor allele** | **Major allele** | **Case (Hom/Het/Wild)** | **Control (Hom/Het/Wild)** | **MAF_AFF** | **MAF_**  **UNAFF** | **MAF gnomAD_**  **genome_EAS** | **P** | **OR (95% CI)** | **Groups** |
| --- | --- | --- | --- | --- | --- | --- | --- | --- | --- | --- | --- |
| 14:55335468 | rs12323905 | T | C | 412/920/475 | 235/582/371 | 0.483 | 0.443 | 0.433 | 0.001 | 1.19(1.07-1.32) | UTRs/introns |
| 14:55336751 | rs2183081 | A | G | 440/931/479 | 246/601/366 | 0.490 | 0.451 | 0.433 | 0.001 | 1.19(1.07-1.32) | UTRs/introns |
| 14:55340856 | rs79159412 | G | A | 438/943/488 | 242/602/367 | 0.487 | 0.448 | 0.431 | 0.002 | 1.18(1.06-1.31) | UTRs/introns |
| 14:55340740 | rs80200535 | T | C | 439/925/482 | 242/594/360 | 0.488 | 0.451 | 0.433 | 0.002 | 1.18(1.06-1.31) | UTRs/introns |
| 14:55336438 | rs2183082 | C | T | 433/961/480 | 250/607/372 | 0.488 | 0.450 | 0.431 | 0.002 | 1.18(1.06-1.31) | UTRs/introns |
| 14:55352521 | rs3783640 | T | C | 442/993/491 | 252/618/373 | 0.487 | 0.451 | 0.428 | 0.002 | 1.17(1.06-1.3) | UTRs/introns |
| 14:55342161 | rs10220344 | T | C | 442/941/482 | 252/588/365 | 0.489 | 0.453 | 0.434 | 0.003 | 1.17(1.06-1.3) | UTRs/introns |
| 14:55335062 | rs2183084 | C | G | 435/936/477 | 256/598/372 | 0.489 | 0.453 | 0.433 | 0.003 | 1.17(1.06-1.3) | UTRs/introns |
| 14:55348869 | rs11158026 | T | C | 433/961/480 | 250/607/372 | 0.488 | 0.450 | 0.433 | 0.003 | 1.17(1.05-1.3) | GH14J054880, GWAS, intron/UTR |
| 14:55352863 | rs10136545 | C | T | 456/957/479 | 267/618/369 | 0.494 | 0.459 | 0.437 | 0.004 | 1.17(1.05-1.29) | UTRs/introns |
| 14:55338650 | rs61039049 | T | C | 269/880/623 | 231/560/378 | 0.400 | 0.437 | 0.447 | 0.004 | 0.85(0.77-0.95) | eQTLs, UTRs/introns |
| 14:55336360 | rs7141319 | G | A | 285/870/689 | 230/560/405 | 0.391 | 0.427 | 0.442 | 0.004 | 0.86(0.77-0.95) | eQTLs, UTRs/introns |
| 14:55353788 | rs28417208 | T | A | 455/961/485 | 261/609/365 | 0.492 | 0.458 | 0.437 | 0.004 | 1.16(1.05-1.29) | UTRs/introns |
| 14:55338321 | rs11850691 | G | A | 278/885/688 | 230/554/410 | 0.389 | 0.425 | 0.445 | 0.005 | 0.86(0.77-0.95) | eQTLs, UTRs/introns |
| 14:55349103 | rs11158027 | C | T | 294/914/691 | 233/588/408 | 0.396 | 0.429 | 0.454 | 0.01 | 0.86(0.78-0.96) | GH14J054880, eQTLs, UTRs/introns |
| 14:55336359 | rs7141483 | T | C | 284/869/687 | 231/560/411 | 0.391 | 0.425 | 0.442 | 0.01 | 0.86(0.78-0.96) | eQTLs, UTRs/introns |
| 14:55346273 | rs10133650 | C | G | 396/928/488 | 228/590/365 | 0.475 | 0.442 | 0.433 | 0.01 | 1.16(1.04-1.29) | UTRs/introns |
| 14:55337939 | rs4363781 | G | A | 282/908/702 | 227/595/422 | 0.389 | 0.422 | 0.443 | 0.01 | 0.87(0.78-0.96) | UTRs/introns |
| 14:55353717 | rs10498471 | A | G | 291/919/691 | 232/589/413 | 0.395 | 0.427 | 0.452 | 0.01 | 0.87(0.78-0.96) | eQTLs, UTRs/introns |
| 14:55348373 | rs3783638 | A | G | 294/905/684 | 231/589/407 | 0.396 | 0.428 | 0.452 | 0.01 | 0.87(0.78-0.96) | GH14J054880, eQTLs, UTRs/introns |
| 14:55341903 | rs140884539 | T | C | 286/890/680 | 228/575/404 | 0.394 | 0.427 | 0.441 | 0.01 | 0.87(0.78-0.96) | eQTLs, UTRs/introns |
| 14:55350666 | rs8004445 | T | G | 295/897/676 | 234/579/405 | 0.398 | 0.430 | 0.453 | 0.01 | 0.87(0.78-0.97) | eQTLs, UTRs/introns |
| 14:55337916 | rs4363780 | G | A | 285/918/698 | 228/589/417 | 0.391 | 0.423 | 0.444 | 0.01 | 0.87(0.78-0.97) | UTRs/introns |
| 14:55337673 | rs10134163 | C | T | 282/917/698 | 223/598/418 | 0.390 | 0.421 | 0.442 | 0.01 | 0.87(0.79-0.97) | eQTLs, UTRs/introns |
| 14:55300378 | rs7148266 | T | C | 199/825/859 | 148/573/498 | 0.325 | 0.356 | 0.357 | 0.01 | 0.87(0.78-0.97) | eQTLs |
| 14:55344015 | rs12147422 | C | T | 299/899/673 | 232/584/399 | 0.400 | 0.431 | 0.453 | 0.01 | 0.87(0.79-0.97) | eQTLs, UTRs/introns |
| 14:55336277 | rs7141433 | T | C | 273/904/722 | 217/587/436 | 0.382 | 0.412 | 0.433 | 0.01 | 0.87(0.79-0.97) | eQTLs, UTRs/introns |
| 14:55348118 | rs3783637 | T | C | 297/859/669 | 232/561/399 | 0.398 | 0.430 | 0.453 | 0.01 | 0.87(0.79-0.97) | GH14J054880, eQTLs, UTRs/introns |
| 14:55337653 | rs10145097 | A | G | 283/920/701 | 226/591/423 | 0.390 | 0.421 | 0.443 | 0.01 | 0.87(0.79-0.97) | eQTLs, UTRs/introns |
| 14:55320785 | rs2878168 | A | G | 201/845/840 | 154/566/490 | 0.331 | 0.361 | 0.360 | 0.01 | 0.87(0.78-0.97) | eQTLs, UTRs/introns |
| 14:55350696 | rs8004018 | G | A | 301/912/676 | 236/588/406 | 0.401 | 0.431 | 0.453 | 0.01 | 0.88(0.79-0.97) | eQTLs, UTRs/introns |
| 14:55310492 | rs841 | A | G | 197/840/857 | 149/575/506 | 0.326 | 0.355 | 0.354 | 0.01 | 0.87(0.78-0.97) | GH14J054843, eQTLs, UTRs/introns |
| 14:55314949 | rs72713467 | T | G | 196/792/824 | 149/553/493 | 0.327 | 0.356 | 0.357 | 0.01 | 0.87(0.78-0.97) | eQTLs, UTRs/introns |
| 14:55337869 | rs4402455 | G | T | 287/908/698 | 228/584/422 | 0.391 | 0.421 | 0.443 | 0.01 | 0.88(0.79-0.97) | eQTLs, UTRs/introns |
| 14:55347827 | rs7155501 | A | G | 424/903/484 | 247/596/355 | 0.483 | 0.455 | 0.432 | 0.02 | 1.14(1.03-1.26) | GH14J054880, UTRs/introns |
| 14:55341720 | rs10140721 | C | A | 282/883/695 | 222/553/415 | 0.389 | 0.419 | 0.442 | 0.02 | 0.88(0.79-0.98) | eQTLs, UTRs/introns |
| 14:55351185 | rs8010461 | G | T | 303/907/674 | 233/592/404 | 0.402 | 0.430 | 0.457 | 0.02 | 0.88(0.79-0.98) | UTRs/introns |
| 14:55322851 | rs7155309 | C | T | 194/827/838 | 150/557/498 | 0.327 | 0.356 | 0.355 | 0.02 | 0.87(0.78-0.98) | eQTLs, UTRs/introns |
| 14:55311569 | rs752688 | T | C | 193/840/860 | 149/572/517 | 0.324 | 0.351 | 0.357 | 0.02 | 0.88(0.79-0.98) | GH14J054843, eQTLs, UTRs/introns |
| 14:55348837 | rs3825611 | G | C | 297/924/684 | 232/592/416 | 0.398 | 0.426 | 0.455 | 0.02 | 0.88(0.79-0.98) | GH14J054880, eQTLs, UTRs/introns |
| 14:55327537 | rs2149483 | A | G | 190/827/846 | 147/545/496 | 0.324 | 0.353 | 0.360 | 0.02 | 0.88(0.78-0.98) | eQTLs, UTRs/introns |
| 14:55294132 | rs58475937 | - | A | 191/828/854 | 143/563/502 | 0.323 | 0.351 | 0.354 | 0.02 | 0.88(0.79-0.98) | eQTLs |
| 14:55344901 | rs28477407 | T | C | 299/912/680 | 229/589/408 | 0.399 | 0.427 | 0.455 | 0.02 | 0.88(0.8-0.98) | eQTLs, UTRs/introns |
| 14:55325862 | rs12589758 | T | A | 192/846/869 | 149/569/522 | 0.323 | 0.350 | 0.354 | 0.02 | 0.88(0.79-0.98) | GH14J054857, eQTLs, UTRs/introns |
| 14:55346373 | rs61702394 | G | A | 298/900/666 | 233/577/406 | 0.401 | 0.429 | 0.453 | 0.02 | 0.88(0.8-0.98) | eQTLs, UTRs/introns |
| 14:55291048 | rs72713456 | C | T | 193/832/855 | 143/561/499 | 0.324 | 0.352 | 0.360 | 0.02 | 0.88(0.79-0.98) | eQTLs |
| 14:55325583 | rs12587434 | G | T | 192/831/862 | 147/564/517 | 0.322 | 0.349 | 0.353 | 0.02 | 0.88(0.79-0.98) | GH14J054857, eQTLs, UTRs/introns |
| 14:55339610 | rs200640299 | G | A | 267/844/688 | 213/522/415 | 0.383 | 0.412 | 0.424 | 0.02 | 0.88(0.79-0.98) | eQTLs, UTRs/introns |
| 14:55369502 | rs1753589 | A | G | 4/157/1790 | 3/74/1189 | 0.042 | 0.032 | 0.028 | 0.02 | 1.37(1.04-1.8) | GH14J054900, UTRs/introns |
| 14:55306457 | rs10483639 | C | G | 194/807/852 | 144/559/511 | 0.323 | 0.349 | 0.358 | 0.03 | 0.88(0.79-0.99) | eQTLs |
| 14:55335747 | rs10138429 | G | A | 286/893/670 | 222/569/405 | 0.396 | 0.424 | 0.447 | 0.03 | 0.89(0.8-0.99) | eQTLs, UTRs/introns |
| 14:55343067 | rs10146658 | T | C | 287/880/666 | 227/564/407 | 0.397 | 0.425 | 0.443 | 0.03 | 0.89(0.8-0.99) | eQTLs, UTRs/introns |
| 14:55320563 | rs4411417 | C | T | 193/809/836 | 146/553/504 | 0.325 | 0.351 | 0.356 | 0.03 | 0.88(0.79-0.99) | eQTLs, UTRs/introns |
| 14:55328814 | rs148193231 | G | A | 0/48/1876 | 0/19/1236 | 0.012 | 0.008 | 0.006 | 0.03 | 1.85(1.07-3.2) | UTRs/introns |
| 14:55353187 | rs10139282 | A | G | 283/860/670 | 221/553/413 | 0.393 | 0.419 | 0.452 | 0.03 | 0.89(0.8-0.99) | eQTLs, UTRs/introns |
| 14:55347834 | rs118144291 | T | C | 23/237/1580 | 8/132/1065 | 0.077 | 0.061 | 0.059 | 0.03 | 1.25(1.03-1.53) | GH14J054880, UTRs/introns |
| 14:55349258 | rs10873086 | T | C | 294/908/680 | 230/573/421 | 0.397 | 0.422 | 0.452 | 0.03 | 0.89(0.8-0.99) | GH14J054880, eQTLs, UTRs/introns |
| 14:55323450 | rs1952437 | T | C | 229/797/843 | 120/529/583 | 0.336 | 0.312 | 0.302 | 0.03 | 1.13(1.01-1.26) | UTRs/introns |
| 14:55304009 | rs2057368 | A | G | 193/824/836 | 150/544/506 | 0.327 | 0.352 | 0.357 | 0.03 | 0.89(0.79-0.99) | eQTLs |
| 14:55336875 | rs7492600 | T | G | 288/889/680 | 216/575/404 | 0.395 | 0.421 | 0.444 | 0.03 | 0.89(0.8-0.99) | eQTLs, UTRs/introns |
| 14:55319514 | rs11626298 | A | G | 228/854/743 | 178/570/451 | 0.359 | 0.386 | 0.390 | 0.03 | 0.89(0.8-0.99) | eQTLs, UTRs/introns |
| 14:55343879 | rs17128050 | C | T | 287/886/693 | 217/582/425 | 0.391 | 0.415 | 0.442 | 0.04 | 0.89(0.81-0.99) | eQTLs, UTRs/introns |
| 14:55306240 | rs17128004 | A | C | 198/837/846 | 146/568/506 | 0.328 | 0.353 | 0.357 | 0.04 | 0.89(0.8-0.99) | eQTLs |
| 14:55290019 | rs55985131 | T | C | 196/842/852 | 148/581/520 | 0.327 | 0.351 | 0.356 | 0.04 | 0.89(0.8-0.99) | eQTLs |
| 14:55318346 | rs7161034 | A | C | 219/890/772 | 170/579/469 | 0.353 | 0.377 | 0.392 | 0.05 | 0.9(0.8-1) | eQTLs, UTRs/introns |
| 14:55316270 | rs10133662 | G | A | 229/882/753 | 176/586/461 | 0.359 | 0.384 | 0.393 | 0.05 | 0.9(0.81-1) | eQTLs, UTRs/introns |
| 14:55315908 | rs10131232 | A | G | 228/903/762 | 180/588/472 | 0.359 | 0.382 | 0.393 | 0.06 | 0.9(0.81-1) | eQTLs, UTRs/introns |
| 14:55315005 | rs11848732 | C | T | 221/859/731 | 173/569/451 | 0.359 | 0.384 | 0.392 | 0.06 | 0.9(0.81-1) | eQTLs, UTRs/introns |
| 14:55316512 | rs10133941 | T | C | 225/886/766 | 173/573/467 | 0.356 | 0.379 | 0.392 | 0.06 | 0.9(0.81-1) | eQTLs, UTRs/introns |
| 14:55328635 | rs9671371 | T | C | 197/822/829 | 147/553/502 | 0.329 | 0.352 | 0.359 | 0.06 | 0.9(0.81-1.01) | eQTLs, UTRs/introns |
| 14:55304080 | rs2145945 | A | G | 229/881/742 | 178/559/450 | 0.362 | 0.385 | 0.390 | 0.06 | 0.9(0.81-1.01) | eQTLs |
| 14:55324848 | rs8007201 | G | A | 233/806/742 | 175/528/449 | 0.357 | 0.381 | 0.381 | 0.06 | 0.9(0.81-1.01) | GH14J054857, eQTLs, UTRs/introns |
| 14:55285160 | rs72713453 | T | C | 199/839/843 | 140/573/502 | 0.329 | 0.351 | 0.356 | 0.07 | 0.9(0.81-1.01) | GH14J054818, eQTLs |
| 14:56209898 | rs34892660 | T | C | 5/231/1652 | 3/126/1098 | 0.064 | 0.054 | 0.072 | 0.07 | 1.23(0.98-1.53) | eQTLs |
| 14:55327553 | rs74797638 | A | G | 83/595/1202 | 58/422/731 | 0.202 | 0.222 | 0.235 | 0.07 | 0.89(0.79-1.01) | UTRs/introns |
| 14:55304061 | rs2057369 | A | G | 196/828/834 | 147/541/504 | 0.328 | 0.350 | 0.356 | 0.07 | 0.9(0.81-1.01) | eQTLs |
| 14:55335570 | rs10138301 | A | G | 3/147/1774 | 2/75/1178 | 0.040 | 0.031 | 0.029 | 0.07 | 1.29(0.98-1.7) | UTRs/introns |
| 14:55329698 | rs76134883 | G | A | 1/57/1815 | 0/51/1162 | 0.016 | 0.021 | 0.013 | 0.08 | 0.71(0.48-1.04) | UTRs/introns |
| 14:55296729 | rs17127992 | G | T | 196/839/859 | 143/558/521 | 0.325 | 0.345 | 0.356 | 0.08 | 0.91(0.81-1.01) | eQTLs |
| 14:55345241 | rs201210131 | - | C | 1/74/1826 | 1/33/1205 | 0.020 | 0.014 | 0.017 | 0.09 | 1.42(0.95-2.13) | UTRs/introns |
| 14:55288924 | rs9323272 | A | G | 224/901/761 | 169/586/463 | 0.358 | 0.379 | 0.392 | 0.09 | 0.91(0.82-1.01) | eQTLs |
| 14:55348412 | rs79272564 | G | A | 2/92/1810 | 2/79/1163 | 0.025 | 0.033 | 0.037 | 0.10 | 0.78(0.58-1.05) | GH14J054880, UTRs/introns |
| 14:55292020 | rs10135789 | G | A | 202/870/797 | 155/568/499 | 0.341 | 0.359 | 0.369 | 0.11 | 0.92(0.82-1.02) | eQTLs |
| 14:55249345 | rs709939 | C | T | 255/841/787 | 160/523/538 | 0.359 | 0.345 | 0.338 | 0.12 | 1.09(0.98-1.21) | GH14J054782 |
| 14:55251933 | rs80195014 | T | G | 2/34/1909 | 0/36/1222 | 0.010 | 0.014 | 0.015 | 0.14 | 0.71(0.45-1.12) | GH14J054782 |
| 14:55326991 | rs76585724 | A | C | 3/102/1799 | 3/50/1192 | 0.028 | 0.022 | 0.024 | 0.14 | 1.27(0.92-1.76) | UTRs/introns |
| 14:55383145 | rs943913 | G | A | 36/446/1430 | 26/309/903 | 0.136 | 0.146 | 0.134 | 0.15 | 0.9(0.78-1.04) | eQTLs |
| 14:55385327 | rs943912 | G | C | 31/408/1418 | 24/283/905 | 0.127 | 0.137 | 0.123 | 0.16 | 0.9(0.77-1.04) | eQTLs |
| 14:55285588 | rs7144602 | G | T | 237/910/749 | 173/586/459 | 0.365 | 0.383 | 0.394 | 0.16 | 0.93(0.83-1.03) | GH14J054818 |
| 14:55348666 | rs3783639 | C | T | 28/355/1532 | 17/258/968 | 0.107 | 0.118 | 0.109 | 0.17 | 0.89(0.76-1.05) | GH14J054880, eQTLs, UTRs/introns |
| 14:55354146 | rs149856065 | AA | - | 25/337/1512 | 18/241/961 | 0.103 | 0.114 | 0.109 | 0.18 | 0.89(0.76-1.05) | eQTLs, UTRs/introns |
| 14:55343374 | rs143884543 | T | C | 2/119/1695 | 1/62/1133 | 0.034 | 0.027 | 0.025 | 0.18 | 1.24(0.91-1.69) | UTRs/introns |
| 14:55375424 | rs767362156 | - | TTG | 1/91/1828 | 1/45/1214 | 0.024 | 0.019 | 0.015 | 0.18 | 1.28(0.89-1.83) | GH14J054908 |
| 14:55397257 | rs55726650 | G | T | 35/414/1435 | 26/285/917 | 0.129 | 0.137 | 0.125 | 0.18 | 0.9(0.78-1.05) | eQTLs |
| 14:55420673 | rs11629355 | T | C | 30/401/1402 | 22/281/899 | 0.126 | 0.135 | 0.122 | 0.19 | 0.9(0.77-1.05) | eQTLs |
| 14:55310723 | rs190993883 | G | A | 2/117/1774 | 0/64/1184 | 0.032 | 0.026 | 0.025 | 0.20 | 1.23(0.9-1.67) | GH14J054843, UTRs/introns |
| 14:55357502 | rs34544088 | A | G | 29/377/1456 | 20/268/920 | 0.117 | 0.128 | 0.118 | 0.21 | 0.9(0.77-1.06) | GH14J054889, UTRs/introns |
| 14:55358665 | rs7147286 | A | G | 193/768/920 | 112/555/561 | 0.307 | 0.317 | 0.317 | 0.21 | 0.93(0.83-1.04) | GH14J054889, UTRs/introns |
| 14:55801687 | rs9323280 | C | A | 33/385/1423 | 20/274/882 | 0.123 | 0.134 | 0.132 | 0.23 | 0.91(0.78-1.06) | eQTLs |
| 14:55249230 | rs971256 | A | G | 345/903/643 | 218/574/433 | 0.421 | 0.412 | 0.400 | 0.24 | 1.06(0.96-1.18) | GH14J054782 |
| 14:55388723 | rs55751189 | A | G | 34/414/1448 | 23/291/937 | 0.127 | 0.135 | 0.123 | 0.24 | 0.91(0.79-1.06) | eQTLs |
| 14:55385723 | rs72715547 | G | A | 36/434/1416 | 24/303/906 | 0.134 | 0.142 | 0.134 | 0.24 | 0.92(0.79-1.06) | eQTLs |
| 14:55358431 | rs77166118 | C | A | 50/518/1326 | 30/362/845 | 0.163 | 0.171 | 0.165 | 0.25 | 0.92(0.8-1.06) | GH14J054889, UTRs/introns |
| 14:55351579 | rs67620272 | C | G | 30/338/1503 | 17/244/944 | 0.106 | 0.115 | 0.110 | 0.25 | 0.91(0.77-1.07) | eQTLs, UTRs/introns |
| 14:55361462 | rs10545051 | - | AA | 29/367/1464 | 16/269/935 | 0.114 | 0.123 | 0.116 | 0.26 | 0.91(0.78-1.07) | UTRs/introns |
| 14:55359690 | rs142520205 | A | G | 1/50/1754 | 0/43/1151 | 0.014 | 0.018 | 0.012 | 0.27 | 0.79(0.53-1.2) | GH14J054889, UTRs/introns |
| 14:55349549 | rs11626210 | T | C | 30/359/1512 | 18/255/953 | 0.110 | 0.119 | 0.111 | 0.28 | 0.92(0.78-1.08) | GH14J054880, eQTLs, UTRs/introns |
| 14:55348429 | rs143104721 | C | T | 2/36/1881 | 0/34/1222 | 0.010 | 0.014 | 0.006 | 0.29 | 0.78(0.5-1.23) | GH14J054880, UTRs/introns |
| 14:55351266 | rs58293795 | C | T | 28/352/1498 | 16/257/970 | 0.109 | 0.116 | 0.109 | 0.29 | 0.92(0.78-1.08) | eQTLs, UTRs/introns |
| 14:55253864 | rs2281652 | G | A | 243/853/791 | 166/525/544 | 0.355 | 0.347 | 0.336 | 0.29 | 1.06(0.95-1.18) | GH14J054782 |
| 14:55411744 | rs17253619 | C | T | 4/85/1812 | 2/70/1177 | 0.024 | 0.030 | 0.032 | 0.30 | 0.85(0.63-1.16) | eQTLs |
| 14:55470098 | rs73266074 | A | G | 4/80/1740 | 3/62/1119 | 0.024 | 0.029 | 0.032 | 0.32 | 0.85(0.62-1.17) | eQTLs |
| 14:55384451 | rs1107961 | G | T | 34/449/1417 | 25/304/908 | 0.136 | 0.143 | 0.134 | 0.32 | 0.93(0.8-1.08) | eQTLs |
| 14:55252085 | rs77373679 | A | G | 0/78/1853 | 0/59/1208 | 0.020 | 0.023 | 0.025 | 0.32 | 0.84(0.59-1.19) | GH14J054782 |
| 14:55353368 | rs8020798 | T | C | 27/352/1498 | 16/250/956 | 0.108 | 0.115 | 0.110 | 0.32 | 0.92(0.78-1.08) | eQTLs, UTRs/introns |
| 14:55352041 | rs72713477 | C | T | 26/362/1525 | 16/256/978 | 0.108 | 0.115 | 0.109 | 0.33 | 0.92(0.79-1.09) | UTRs/introns |
| 14:55252318 | rs199837694 | - | A | 0/46/1891 | 0/23/1237 | 0.012 | 0.009 | 0.008 | 0.34 | 1.28(0.77-2.14) | GH14J054782 |
| 14:55249936 | rs201231744 | - | CTT | 0/74/1813 | 1/54/1184 | 0.020 | 0.023 | 0.023 | 0.35 | 0.84(0.59-1.2) | GH14J054782 |
| 14:55382478 | rs76048263 | T | - | 32/425/1394 | 25/284/895 | 0.132 | 0.139 | 0.133 | 0.35 | 0.93(0.8-1.08) | eQTLs |
| 14:55368353 | rs140395506 | C | G | 1/53/1872 | 0/29/1223 | 0.014 | 0.012 | 0.012 | 0.35 | 1.24(0.79-1.95) | GH14J054900, UTRs/introns |
| 14:55251012 | rs541479660) | G | - | 2/44/1861 | 0/39/1209 | 0.013 | 0.016 | 0.017 | 0.35 | 0.82(0.54-1.25) | GH14J054782 |
| 14:55357819 | rs10645822 | TTTG | - | 31/358/1453 | 17/260/934 | 0.114 | 0.121 | 0.116 | 0.35 | 0.93(0.79-1.09) | GH14J054889, UTRs/introns |
| 14:55375602 | rs11621177 | A | C | 34/429/1385 | 24/290/883 | 0.135 | 0.141 | 0.135 | 0.35 | 0.93(0.8-1.08) | eQTLs |
| 14:55375426 | rs11621165 | A | G | 38/459/1337 | 26/313/861 | 0.146 | 0.152 | 0.136 | 0.36 | 0.93(0.81-1.08) | GH14J054908 |
| 14:55351419 | rs138478970 | - | C | 26/343/1430 | 17/242/924 | 0.110 | 0.117 | 0.108 | 0.36 | 0.93(0.79-1.09) | eQTLs, UTRs/introns |
| 14:55365786 | rs60258834 | A | C | 36/436/1449 | 20/305/921 | 0.132 | 0.138 | 0.120 | 0.36 | 0.93(0.8-1.08) | UTRs/introns |
| 14:55297043 | rs72713460 | T | G | 30/350/1421 | 19/245/915 | 0.114 | 0.120 | 0.120 | 0.36 | 0.93(0.79-1.09) | eQTLs |
| 14:55358877 | rs7147201 | G | A | 30/378/1461 | 18/266/933 | 0.117 | 0.124 | 0.117 | 0.37 | 0.93(0.79-1.09) | GH14J054889, UTRs/introns |
| 14:55360836 | rs72713482 | A | T | 28/348/1500 | 17/243/952 | 0.108 | 0.114 | 0.106 | 0.37 | 0.93(0.79-1.09) | eQTLs, UTRs/introns |
| 14:55252734 | rs75275673 | A | G | 0/78/1845 | 0/58/1199 | 0.020 | 0.023 | 0.024 | 0.37 | 0.85(0.6-1.21) | GH14J054782 |
| 14:55370285 | rs10136972 | G | A | 52/500/1348 | 33/344/866 | 0.159 | 0.165 | 0.162 | 0.37 | 0.94(0.82-1.08) | GH14J054900 |
| 14:55360139 | rs3783641 | A | T | 30/384/1490 | 17/271/945 | 0.117 | 0.124 | 0.117 | 0.38 | 0.93(0.8-1.09) | GH14J054889, UTRs/introns |
| 14:55338340 | rs11627828 | T | C | 31/367/1429 | 19/259/928 | 0.117 | 0.123 | 0.121 | 0.38 | 0.93(0.8-1.09) | eQTLs, UTRs/introns |
| 14:55370577 | rs112756830 | T | - | 45/429/1296 | 33/288/834 | 0.147 | 0.153 | 0.157 | 0.38 | 0.94(0.81-1.08) | GH14J054900 |
| 14:55252907 | rs146591022 | A | G | 0/49/1846 | 0/28/1203 | 0.013 | 0.011 | 0.007 | 0.39 | 1.23(0.77-1.99) | GH14J054782 |
| 14:55342077 | rs11626155 | T | C | 30/384/1476 | 17/265/934 | 0.118 | 0.123 | 0.123 | 0.39 | 0.93(0.8-1.09) | eQTLs, UTRs/introns |
| 14:55360203 | rs3783642 | C | T | 201/780/909 | 114/551/558 | 0.313 | 0.319 | 0.320 | 0.40 | 0.95(0.85-1.07) | GH14J054889, UTRs/introns |
| 14:55357771 | rs8003903 | T | C | 68/569/1246 | 41/384/791 | 0.187 | 0.192 | 0.200 | 0.40 | 0.95(0.83-1.08) | GH14J054889, eQTLs, UTRs/introns |
| 14:55340251 | rs201085378 | A | - | 24/361/1434 | 15/254/931 | 0.112 | 0.118 | 0.120 | 0.41 | 0.93(0.79-1.1) | eQTLs, UTRs/introns |
| 14:55365381 | rs8018688 | G | A | 36/414/1478 | 18/295/942 | 0.126 | 0.132 | 0.120 | 0.41 | 0.94(0.81-1.09) | UTRs/introns |
| 14:55254275 | rs2281653 | G | A | 332/918/636 | 222/578/433 | 0.419 | 0.414 | 0.399 | 0.43 | 1.04(0.94-1.16) | GH14J054782 |
| 14:55254399 | rs147345486 | T | C | 0/71/1849 | 0/40/1206 | 0.018 | 0.016 | 0.015 | 0.43 | 1.18(0.79-1.75) | GH14J054782 |
| 14:55343379 | rs186160820 | T | C | 1/60/1747 | 0/35/1148 | 0.017 | 0.015 | 0.022 | 0.43 | 1.19(0.78-1.81) | UTRs/introns |
| 14:55366683 | rs185480698 | T | A | 1/71/1798 | 0/40/1172 | 0.020 | 0.017 | 0.014 | 0.43 | 1.17(0.79-1.74) | UTRs/introns |
| 14:55252971 | rs76383762 | T | C | 0/80/1802 | 0/58/1169 | 0.021 | 0.024 | 0.024 | 0.44 | 0.87(0.61-1.24) | GH14J054782 |
| 14:55351179 | rs56013432 | C | T | 31/355/1499 | 18/250/969 | 0.111 | 0.116 | 0.109 | 0.45 | 0.94(0.8-1.1) | UTRs/introns |
| 14:55359653 | rs113585929 | T | C | 66/540/1190 | 41/358/752 | 0.187 | 0.191 | 0.194 | 0.45 | 0.95(0.83-1.09) | GH14J054889, eQTLs, UTRs/introns |
| 14:55363321 | rs10131633 | A | G | 189/780/912 | 109/544/572 | 0.308 | 0.311 | 0.318 | 0.45 | 0.96(0.86-1.07) | UTRs/introns |
| 14:55445126 | rs75790544 | A | C | 4/84/1735 | 1/68/1133 | 0.025 | 0.029 | 0.030 | 0.45 | 0.89(0.65-1.21) | eQTLs |
| 14:55253071 | rs138814133 | T | A | 4/78/1839 | 2/47/1201 | 0.022 | 0.020 | 0.015 | 0.46 | 1.14(0.81-1.61) | GH14J054782 |
| 14:55365952 | rs56213135 | T | G | 36/422/1422 | 20/295/912 | 0.131 | 0.137 | 0.120 | 0.46 | 0.95(0.81-1.1) | UTRs/introns |
| 14:55386646 | rs8011358 | C | T | 35/407/1432 | 25/268/921 | 0.127 | 0.131 | 0.123 | 0.47 | 0.95(0.81-1.1) | eQTLs |
| 14:55301494 | rs57875940 | A | G | 30/356/1456 | 16/251/936 | 0.113 | 0.118 | 0.121 | 0.50 | 0.95(0.81-1.11) | eQTLs |
| 14:55327997 | rs149850176 | A | T | 2/80/1802 | 0/48/1161 | 0.022 | 0.020 | 0.030 | 0.51 | 1.13(0.79-1.62) | UTRs/introns |
| 14:55374601 | rs11847771 | A | T | 41/440/1396 | 25/298/893 | 0.139 | 0.143 | 0.136 | 0.51 | 0.95(0.82-1.1) | eQTLs |
| 14:55370680 | rs3759663 | A | C | 30/391/1450 | 17/267/935 | 0.121 | 0.124 | 0.109 | 0.51 | 0.95(0.81-1.11) | GH14J054900 |
| 14:55316722 | rs147772763 | C | T | 2/65/1844 | 0/40/1208 | 0.018 | 0.016 | 0.023 | 0.53 | 1.14(0.77-1.68) | UTRs/introns |
| 14:55347786 | rs111537263 | A | G | 27/338/1457 | 15/236/940 | 0.108 | 0.112 | 0.108 | 0.53 | 0.95(0.8-1.12) | GH14J054880, eQTLs, UTRs/introns |
| 14:55364169 | rs112159692 | A | G | 66/539/1217 | 42/354/776 | 0.184 | 0.187 | 0.199 | 0.53 | 0.96(0.84-1.1) | eQTLs, UTRs/introns |
| 14:55338256 | rs11627767 | G | A | 32/382/1472 | 19/263/950 | 0.118 | 0.122 | 0.122 | 0.54 | 0.95(0.81-1.11) | eQTLs, UTRs/introns |
| 14:55355269 | rs149220062 | T | C | 1/53/1768 | 0/42/1156 | 0.015 | 0.018 | 0.017 | 0.56 | 0.88(0.59-1.33) | GH14J054889, UTRs/introns |
| 14:55356525 | rs17128052 | C | G | 27/355/1508 | 15/245/973 | 0.108 | 0.112 | 0.108 | 0.57 | 0.95(0.81-1.12) | GH14J054889, eQTLs, UTRs/introns |
| 14:55343107 | rs60851395 | A | G | 31/388/1474 | 18/261/931 | 0.119 | 0.123 | 0.122 | 0.58 | 0.96(0.82-1.12) | eQTLs, UTRs/introns |
| 14:55361836 | rs8017210 | A | G | 32/379/1484 | 17/263/951 | 0.117 | 0.121 | 0.116 | 0.58 | 0.96(0.82-1.12) | UTRs/introns |
| 14:55285069 | rs138879443 | A | G | 0/63/1852 | 1/46/1197 | 0.016 | 0.019 | 0.030 | 0.59 | 0.9(0.61-1.32) | GH14J054818 |
| 14:55359664 | rs112165923 | C | G | 66/544/1207 | 39/363/769 | 0.186 | 0.188 | 0.194 | 0.59 | 0.96(0.84-1.1) | GH14J054889, eQTLs, UTRs/introns |
| 14:55370340 | rs10137071 | T | C | 52/490/1319 | 32/330/849 | 0.160 | 0.163 | 0.162 | 0.60 | 0.96(0.84-1.11) | GH14J054900 |
| 14:55339808 | rs370274640 | A | G | 0/52/1803 | 0/30/1179 | 0.014 | 0.012 | 0.013 | 0.60 | 1.13(0.71-1.79) | UTRs/introns |
| 14:55357742 | rs57095876 | G | A | 26/362/1512 | 14/249/975 | 0.109 | 0.112 | 0.108 | 0.60 | 0.96(0.81-1.13) | GH14J054889, eQTLs, UTRs/introns |
| 14:55320197 | rs17128021 | A | G | 4/92/1783 | 1/60/1166 | 0.027 | 0.025 | 0.027 | 0.60 | 1.09(0.79-1.5) | UTRs/introns |
| 14:55374303 | rs113334991 | C | T | 2/58/1860 | 1/43/1208 | 0.016 | 0.018 | 0.025 | 0.61 | 0.91(0.62-1.33) | eQTLs |
| 14:55455015 | rs17832281 | C | T | 3/90/1795 | 1/68/1169 | 0.025 | 0.028 | 0.032 | 0.61 | 0.92(0.67-1.26) | eQTLs |
| 14:55347916 | rs3825610 | A | T | 28/342/1494 | 16/235/963 | 0.107 | 0.110 | 0.109 | 0.62 | 0.96(0.81-1.13) | GH14J054880, UTRs/introns |
| 14:55385498 | rs72715546 | A | G | 3/65/1833 | 1/40/1201 | 0.019 | 0.017 | 0.029 | 0.63 | 1.1(0.75-1.6) | eQTLs |
| 14:55370620 | rs3759662 | A | G | 34/391/1384 | 18/262/877 | 0.127 | 0.129 | 0.111 | 0.67 | 0.97(0.83-1.13) | GH14J054900, eQTLs |
| 14:55446007 | rs141057912 | G | - | 3/89/1793 | 1/65/1164 | 0.025 | 0.027 | 0.032 | 0.68 | 0.94(0.68-1.29) | eQTLs |
| 14:55337508 | rs190703124 | C | G | 0/43/1890 | 0/30/1214 | 0.011 | 0.012 | 0.010 | 0.68 | 0.91(0.56-1.46) | UTRs/introns |
| 14:55309841 | rs145762799 | - | AAG | 2/86/1793 | 1/54/1162 | 0.024 | 0.023 | 0.028 | 0.70 | 1.07(0.76-1.5) | UTRs/introns |
| 14:55343344 | rs59750960 | G | T | 31/374/1465 | 16/254/940 | 0.117 | 0.118 | 0.121 | 0.70 | 0.97(0.83-1.14) | eQTLs, UTRs/introns |
| 14:55368627 | rs2149482 | T | C | 50/511/1359 | 32/332/870 | 0.159 | 0.161 | 0.159 | 0.70 | 0.97(0.85-1.12) | GH14J054900, UTRs/introns |
| 14:55374309 | rs112700866 | T | C | 3/58/1851 | 1/43/1197 | 0.017 | 0.018 | 0.025 | 0.70 | 0.93(0.64-1.36) | eQTLs |
| 14:55285443 | rs117713259 | A | G | 3/94/1810 | 1/61/1178 | 0.026 | 0.025 | 0.029 | 0.71 | 1.06(0.77-1.46) | GH14J054818 |
| 14:55340311 | rs146123422 | A | G | 24/372/1427 | 13/252/918 | 0.115 | 0.118 | 0.118 | 0.71 | 0.97(0.82-1.14) | UTRs/introns |
| 14:55198024 | rs4901534 | A | G | 39/530/1319 | 33/323/868 | 0.161 | 0.159 | 0.165 | 0.72 | 1.03(0.89-1.18) | eQTLs |
| 14:55458234 | rs73266060 | C | T | 4/90/1788 | 1/67/1166 | 0.026 | 0.028 | 0.032 | 0.72 | 0.94(0.69-1.29) | eQTLs |
| 14:55359169 | rs372805219 | A | G | 1/35/1888 | 0/28/1234 | 0.010 | 0.011 | 0.016 | 0.73 | 0.92(0.56-1.5) | GH14J054889, UTRs/introns |
| 14:55339327 | rs58992937 | T | C | 31/380/1445 | 18/252/924 | 0.119 | 0.121 | 0.122 | 0.73 | 0.97(0.83-1.14) | UTRs/introns |
| 14:55362528 | rs79511230 | A | G | 11/261/1636 | 11/164/1066 | 0.074 | 0.075 | 0.074 | 0.74 | 0.97(0.8-1.17) | UTRs/introns |
| 14:55318127 | rs145283879 | G | A | 22/368/1482 | 17/232/978 | 0.110 | 0.108 | 0.096 | 0.76 | 1.03(0.87-1.21) | UTRs/introns |
| 14:55316743 | rs145304083 | C | A | 0/53/1867 | 1/34/1219 | 0.014 | 0.014 | 0.012 | 0.78 | 0.94(0.61-1.45) | UTRs/introns |
| 14:55349282 | rs75276929 | C | T | 12/224/1677 | 4/150/1094 | 0.065 | 0.063 | 0.073 | 0.79 | 1.03(0.84-1.27) | GH14J054880, UTRs/introns |
| 14:55365331 | rs8019824 | A | T | 49/499/1344 | 31/330/874 | 0.158 | 0.159 | 0.158 | 0.80 | 0.98(0.85-1.13) | UTRs/introns |
| 14:55250942 | rs2236298 | G | A | 5/148/1746 | 4/100/1136 | 0.042 | 0.044 | 0.037 | 0.80 | 0.97(0.75-1.24) | GH14J054782 |
| 14:55448675 | rs73266045 | A | T | 2/90/1800 | 1/63/1177 | 0.025 | 0.026 | 0.032 | 0.81 | 0.96(0.7-1.33) | eQTLs |
| 14:55250505 | rs28648747 | G | C | 5/150/1749 | 4/96/1150 | 0.042 | 0.042 | 0.036 | 0.82 | 1.03(0.8-1.33) | GH14J054782 |
| 14:55368986 | rs2183080 | C | G | 4/97/1836 | 1/63/1193 | 0.027 | 0.026 | 0.039 | 0.84 | 1.03(0.76-1.41) | GH14J054900, eQTLs, UTRs/introns |
| 14:55252614 | rs2281651 | T | C | 397/935/561 | 242/619/371 | 0.457 | 0.448 | 0.475 | 0.84 | 1.01(0.91-1.12) | GH14J054782 |
| 14:55306804 | rs7142517 | A | C | 137/726/1004 | 83/465/639 | 0.268 | 0.266 | 0.272 | 0.84 | 0.99(0.88-1.11) | eQTLs |
| 14:55311979 | rs7493025 | C | T | 5/107/1722 | 1/74/1124 | 0.032 | 0.032 | 0.035 | 0.84 | 1.03(0.77-1.38) | GH14J054843, UTRs/introns |
| 14:55362358 | rs188265206 | C | T | 0/52/1835 | 1/31/1188 | 0.014 | 0.014 | 0.012 | 0.85 | 1.04(0.67-1.62) | UTRs/introns |
| 14:55386034 | rs17128077 | T | C | 2/67/1853 | 1/43/1213 | 0.018 | 0.018 | 0.028 | 0.86 | 1.04(0.71-1.51) | eQTLs |
| 14:55328922 | rs75148669 | T | A | 1/85/1722 | 1/57/1114 | 0.024 | 0.025 | 0.028 | 0.89 | 0.98(0.7-1.37) | UTRs/introns |
| 14:55329920 | rs183457709 | A | G | 2/59/1816 | 2/36/1186 | 0.017 | 0.016 | 0.019 | 0.90 | 1.03(0.69-1.52) | UTRs/introns |
| 14:55329922 | rs188802750 | A | G | 2/59/1833 | 2/36/1197 | 0.017 | 0.016 | 0.019 | 0.90 | 1.03(0.69-1.52) | UTRs/introns |
| 14:55317306 | rs150180569 | A | G | 2/92/1794 | 3/57/1174 | 0.025 | 0.026 | 0.028 | 0.91 | 1.02(0.74-1.4) | UTRs/introns |
| 14:55339016 | rs56091146 | A | G | 29/372/1457 | 16/246/946 | 0.116 | 0.115 | 0.123 | 0.93 | 0.99(0.85-1.17) | eQTLs, UTRs/introns |
| 14:55314213 | rs10139369 | A | T | 2/91/1782 | 1/61/1169 | 0.025 | 0.026 | 0.028 | 0.94 | 1.01(0.73-1.4) | UTRs/introns |
| 14:55311969 | rs193173177 | A | G | 3/85/1789 | 1/58/1167 | 0.024 | 0.024 | 0.027 | 0.94 | 1.01(0.73-1.41) | GH14J054843, UTRs/introns |
| 14:55326667 | rs147071318 | - | TATAAG | 2/91/1820 | 1/62/1192 | 0.025 | 0.026 | 0.027 | 0.95 | 1.01(0.73-1.4) | UTRs/introns |
| 14:55333867 | rs117440296 | A | G | 0/86/1814 | 0/54/1191 | 0.023 | 0.022 | 0.026 | 0.95 | 0.99(0.7-1.41) | UTRs/introns |
| 14:55338607 | rs190476722 | G | A | 0/50/1832 | 1/31/1206 | 0.013 | 0.013 | 0.012 | 0.95 | 0.99(0.63-1.54) | UTRs/introns |
| 14:55385153 | rs72715545 | T | C | 3/64/1860 | 1/44/1205 | 0.018 | 0.018 | 0.026 | 0.96 | 0.99(0.68-1.43) | eQTLs |
| 14:55368079 | rs150565713 | T | - | 4/95/1822 | 2/62/1182 | 0.027 | 0.026 | 0.037 | 0.98 | 1(0.74-1.37) | GH14J054900, eQTLs, UTRs/introns |
| 14:55249892 | rs59182763 | C | A | 4/144/1738 | 3/96/1145 | 0.040 | 0.041 | 0.036 | 0.99 | 1(0.77-1.29) | GH14J054782 |
| 14:55315675 | rs10143089 | C | T | 2/90/1821 | 1/60/1186 | 0.025 | 0.025 | 0.027 | 1.00 | 1(0.72-1.39) | UTRs/introns |
| 14:55370121 | rs66989494 | T | C | 4/96/1832 | 1/64/1182 | 0.027 | 0.026 | 0.039 | 1.00 | 1(0.73-1.36) | GH14J054900, eQTLs |
