# Supplementary material for: GCH1 variants contribute to the risk and earlier age-at-onset of Parkinson’s disease: a two-cohort case-control study
Source: Transl Neurodegener. 2020 Aug 4;9:31. doi: 10.1186/s40035-020-00212-3 (PMC7401216; doi:10.1186/s40035-020-00212-3)
Supplement: Supplementary file 4 — Additional file 4 Table S4. Summary of clinical features of the Parkinson’s disease patients in this study. [file 40035_2020_212_MOESM4_ESM.docx]

**Table S4. Summary of clinical features of the Parkinson's disease patients in this study.**

| **Clinical features** | **Cohort WES** | **Cohort WGS** |
| --- | --- | --- |
| Age at onset | 46.0±8.25 | 61.9±6.94 |
| Age at assessment | 52.3±8.93 | 1.5±0.50 |
| Disease duration | 6.3±5.18 | 66.8±7.07 |
| UPDRS-Part I | 2.4±2.08 | 2.6±2.08 |
| UPDRS-Part II | 12.0±6.87 | 12.3±6.37 |
| UPDRS-Part III | 27.5±15.97 | 27.8±14.06 |
| Tremor score | 3.8±3.77 | 3.5±3.42 |
| Stiffness score | 5.6±4.25 | 5.6±4.13 |
| Bradykinesia score | 10.2±6.67 | 10.2±6.11 |
| Postural instability score | 4.1±3.20 | 4.5±2.94 |
| Hoeh and Yahr stage | 2.2±0.85 | 2.0±0.74 |
| Dyskinesia | 16.31% | 9.92% |
| Freezing gait | 27.45% | 23.66% |
| Motor subtype |  |  |
| Tremor-dominant | 26.56% | 21.70% |
| Intermediate | 17.36% | 16.04% |
| PIGD-dominant | 56.08% | 62.26% |
| MMSE | 27.0±3.19 | 25.6±4.39 |
| PDSS | 115.8±29.67 | 112.3±29.17 |
| RBDQ-HK | 13.4±16.01 | 16.0±17.29 |
| ESS | 7.4±6.08 | 8.4±6.49 |
| HAMD | 5.9±5.60 | 6.1±5.47 |
| HRS | 19.9±6.36 | 18.7±6.92 |
| PFS | 44.2±18.95 | 46.5±19.13 |
| SCOPA-AUT | 7.4±6.88 | 9.5±7.23 |

Results are from linear or logistic regression analyses adjusting for age, sex, BMI, and ancestry. UPDRS = Unified Parkinson’s disease rating scale; MMSE= Mini-mental state examination; PDSS = Parkinson's disease sleep scale; RBDQ-HK = Rapid eyes movement sleep behavior disorder questionnaire-Hong Kong; ESS = Epworth sleepiness scale; HAMD = 17-item Hamilton depression rating Scale; HRS = Hyposmia rating Scale; PFS = Parkinson's disease fatigue scale; SCOPA-AUT = Scales for outcomes in Parkinson’s disease-autonomic; PDQ39 = The 39-item Parkinson’s disease Questionnaire; PFS = Parkinson's disease fatigue scale.
