# Supplementary material for: GCH1 variants contribute to the risk and earlier age-at-onset of Parkinson’s disease: a two-cohort case-control study
Source: Transl Neurodegener. 2020 Aug 4;9:31. doi: 10.1186/s40035-020-00212-3 (PMC7401216; doi:10.1186/s40035-020-00212-3)
Supplement: Supplementary file 5 — Additional file 5 Table S5. Coding variants of GCH1 identified in two cohorts. [file 40035_2020_212_MOESM5_ESM.docx]

**Table S5: Coding variants of *GCH1* identified in two cohorts.**

| **Position (hg19)** | **SNP_ID** | **Exonic Function** | **Nucleotide change** | **Amino acid alteration** | **Cohort WES** | | **Cohort WGS** | | **CADD/ReVe** | **MAF ^a^** | **Pathogenicity** |
| --- | --- | --- | --- | --- | --- | --- | --- | --- | --- | --- | --- |
|  |  |  |  |  | **Case (n=1555)** | **Control (n=2234)** | **Case (n=1962)** | **Control (n=1279)** |  |  |  |
| chr14:55310743 | - | frameshift deletion | c.745delA | p.R249Gfs*76 | 1 | 0 | 0 | 0 | -/- | -/-/- | Reported in DRD [34] |
| chr14:55310762 | - | synonymous | c.726A>G | p.E242E | 0 | 0 | 1 | 0 | -/- | -/-/- | - |
| chr14:55310795 | - | missense | c.693G>C | p.L231F | 0 | 1 | 0 | 0 | 26.5/0.941 | -/-/- | Unreported |
| chr14:55310810 | - | synonymous | c.678G>C | p.V226V | 1 | 0 | 0 | 0 | -/- | -/-/- | - |
| chr14:55312504 | - | missense | c.608G>A | p.G203E | 1 | 0 | 0 | 0 | 28.3/0.959 | -/-/- | Reported in DRD [35] |
| chr14:55312519 | rs201238926 | missense | c.593G>A | p.R198Q | 1 | 0 | 0 | 0 | 9.491/0.315 | 0/-/- | Reported in PD [10] |
| chr14:55312530 | rs199836777 | synonymous | c.582G>A | p.T194T | 0 | 0 | 1 | 0 | -/- | 0/0/- | - |
| chr14:55312533 | - | missense | c.579C>G | p.I193M | 2 | 0 | 0 | 0 | 24.6/0.838 | -/-/- | Unreported |
| chr14:55312539 | - | synonymous | c.573A>G | p.V191V | 0 | 3 | 1 | 1 | -/- | 0.0002/-/0.000457 | - |
| chr14:55312550 | - | stopgain | c.562C>T | p.Q188X | 1 | 0 | 0 | 0 | 37/0.83 | -/-/- | Reported in DRD [36] |
| chr14:55312560 | - | synonymous | c.552C>A | p.R184R | 1 | 0 | 0 | 0 | -/- | -/-/- | - |
| chr14:55326401 | rs150158277 | synonymous | c.507G>A | p.A169A | 0 | 1 | 0 | 0 | -/- | 0/0/- | - |
| chr14:55326450 | - | missense | c.458A>C | p.H153P | 1 | 0 | 0 | 0 | 27/0.999 | -/-/- | Reported in DRD [37] |
| chr14:55332089 | - | missense | c.409A>G | p.M137V | 1 | 0 | 0 | 0 | 9.357/0.93 | -/-/- | Reported in PD [10] |
| chr14:55332135 | - | frameshift insertion | c.362dupT | p.F122Ifs*1 | 1 | 0 | 0 | 0 | -/- | -/-/- | Unreported |
| chr14:55369078 | - | missense | c.304A>T | p.M102L | 1 | 0 | 0 | 0 | 21.6/0.794 | -/-/- | Unreported |
| chr14:55369088 | rs765416856 | synonymous | c.294G>T | p.A98A | 2 | 1 | 0 | 1 | -/- | 0.0007/-/0.000372 | - |
| chr14:55369123 | - | frameshift deletion | c.259delC | p.Q87Sfs*29 | 1 | 0 | 0 | 0 | -/- | -/-/- | Unreported |
| chr14:55369126 | - | missense | c.256C>T | p.P86S | 0 | 0 | 1 | 0 | 23.3/0.795 | -/-/- | Unreported |
| chr14:55369136 | - | synonymous | c.246G>T | p.L82L | 0 | 0 | 1 | 0 | -/- | -/-/- | - |
| chr14:55369143 | rs770547722 | missense | c.239G>A | p.S80N | 4 | 1 | 6 | 2 | 7.538/0.353 | 0.0012/0.0012/0.000742 | Reported in DRD [38] |
| chr14:55369152 | rs748666093 | missense | c.230C>G | p.S77C | 2 | 1 | 3 | 1 | 17.81/0.633 | 0.00005825/-/0.000371 | Reported in PD [10] |
| chr14:55369172 | rs763168809 | missense | c.210C>A | p.N70K | 0 | 0 | 0 | 2 | 1.068/0.337 | 0.0001/-/0.00074 | Unreported |
| chr14:55369186 | - | synonymous | c.196C>T | p.L66L | 0 | 1 | 2 | 3 | -/- | 0.0004/-/0.001112 | - |
| chr14:55369189 | - | stopgain | c.193G>T | p.E65X | 1 | 0 | 0 | 0 | 35/0.491 | -/-/- | Reported in DRD [36] |
| chr14:55369212 | rs756782285 | missense | c.170G>A | p.R57Q | 1 | 0 | 0 | 0 | 19.09/0.501 | 0/-/- | Reported in PD [10] |
| chr14:55369216 | - | missense | c.166G>A | p.E56K | 0 | 0 | 0 | 1 | 19.83/0.568 | -/-/0.00037 | Unreported |
| chr14:55369266 | - | missense | c.116C>T | p.P39L | 0 | 0 | 1 | 0 | 6.882/0.389 | -/-/- | Unreported |
| chr14:55369271 | - | synonymous | c.111G>A | p.E37E | 0 | 0 | 1 | 0 | -/- | -/-/- | - |
| chr14:55369291 | - | missense | c.91G>A | p.G31R | 0 | 1 | 0 | 1 | 13.76/0.399 | -/-/0.000372 | Unreported |
| chr14:55369295 | - | synonymous | c.87G>T | p.R29R | 1 | 0 | 0 | 0 | -/- | -/-/- | - |
| chr14:55369307 | - | synonymous | c.75G>C | p.R25R | 1 | 0 | 0 | 0 | -/- | -/-/- | - |
| chr14:55369314 | rs41298432 | missense | c.68C>T | p.P23L | 0 | 1 | 0 | 0 | 0.014/0.79 | 0/0/- | Reported to be benign [8] |
| chr14:55369350 | - | missense | c.32A>C | p.E11A | 0 | 0 | 1 | 0 | 0.83/0.532 | -/0.0006/- | Unreported |
| Total |  |  |  |  | 25 | 11 | 19 | 12 |  |  |  |

a. Variants minor allele frequencies from gnomAD_genome_EAS, gnomAD_exome_EAS and control of cohort WGS.
